# Multimodal AI reading of genetic complexity and residual marrow composition in acute promyelocytic leukemia

**DOI:** 10.3389/fimmu.2026.1933105

**Published:** 2026-08-24

**Authors:** Minjie Gao, Guifang Ouyang, Yangguang Liu, Shikun Chen

**Affiliations:** 1 Department of Hematology, The First Affiliated Hospital of Ningbo University, Ningbo, Zhejiang, China; 2 College of Artificial Intelligence, Ningbo University of Finance and Economics, Ningbo, Zhejiang, China

**Keywords:** acute promyelocytic leukemia, bone marrow cytomorphology, differentiation syndrome, interphase FISH, karyotype, multimodal deep learning, PML::RARA, residual marrow composition

## Abstract

**Introduction:**

Acute promyelocytic leukemia (APL) is defined by its genetics, yet interphase fluorescence *in situ* hybridization (FISH) for the PML::RARA fusion, the chromosomal complement seen on a metaphase spread, and the marrow’s cellular composition on a Wright–Giemsa smear are read as separate, unconnected examinations. We did not identify a published model trained on two or more of these image types in the same patients with APL.

**Methods:**

We describe two image-derived readouts for a retrospective cohort of patients with APL. A genetic complexity axis classifies the FISH fusion signal pattern and flags any additional cytogenetic abnormality (ACA) beyond the t(15;17), directly from raw, unarranged metaphase spreads. A marrow composition axis extends a cytomorphology pipeline, built on frozen DinoBloom embeddings and a per-cell classification head directly supervised on an annotated subset, from promyelocyte detection to a seven-class differential count. From this composition, we compute a residual hematopoiesis index, the model-estimated fraction of non-leukemic nucleated marrow cells, so that a higher value indicates a larger non-leukemic fraction. The index is a morphology-derived surrogate for residual non-leukemic marrow composition and carries no immune-phenotypic information. The association between the two axes is computed directly from patient-level, out-of-fold branch outputs; a small, three-node fusion module over the two genetic and one composition readout supports only a secondary, exploratory analysis, reported in the Supplementary Material. The cohort held 700 patients with smear, FISH, and karyotype linked per patient, of whom 650 had complete clinical annotation; we evaluated each branch with patient-stratified fivefold cross-validation, five repeats, pooled out-of-fold, and report bootstrap confidence intervals (CIs) over patients. Diagnostic flow cytometry was retrievable for 118 patients, and 80 slides were re-scanned with fields independently re-selected, to check the index against an external measurement and against itself.

**Results:**

The fusion-pattern classifier had a macro-averaged F1 of 0.82 across the five signal classes, and the karyotype ACA reader had an area under the curve (AUC) of 0.86. The residual hematopoiesis index had a median of 0.42 (interquartile range, IQR, 0.29 to 0.58) across the cohort, tracked the flow-derived non-blast fraction at a Spearman correlation of 0.80 (95% CI 0.72 to 0.86) in the 118 patients with flow data, and had an intraclass correlation of 0.91 on re-scanned slides. Genetic complexity and the residual hematopoiesis index were associated at a Spearman *ρ* of 0.24 (95% CI 0.17 to 0.31, *p <* 0.0001), the primary finding of this study, with higher genetic complexity accompanying a larger non-leukemic fraction. The association held under adjustment for metaphase yield, scorable nuclei per FISH image, age, presenting white-cell count, and accession year, with partial correlations between 0.19 and 0.24, and patients with an expert-defined additional abnormality had a higher index than those with the isolated translocation (median 0.46 against 0.41). An exploratory test of the same readouts against the Sanz risk group and DS occurrence, run on the clinically annotated subcohort, showed performance no better than chance, and an adjusted logistic model bounded any effect of an additional cytogenetic abnormality on DS at an odds ratio of 1.07 (95% CI 0.74 to 1.55), in line with two prior clinical cohorts that reported no association between cytogenetic abnormality and DS.

**Conclusion:**

Image-derived genetic complexity was positively associated with the residual hematopoiesis index, and the association persisted under the adjustments and substitutions applied to it. These findings describe a cross-sectional association in a single-center, retrospective cohort, not a validated diagnostic or risk tool. The residual hematopoiesis index summarizes morphology alone, its agreement with flow cytometry is supportive rather than confirmatory, and the secondary DS analysis drew on a smaller, clinically annotated subset of the cohort rather than on every patient.

## 1 Introduction

### 1.1 The genetic diagnostic axis of acute promyelocytic leukemia

Acute promyelocytic leukemia (APL) is a subtype of acute myeloid leukemia (AML) driven by the PML::RARA fusion, usually from the t(15;17) translocation but sometimes from a cryptic or variant rearrangement, and it is unusual among leukemias in being curable, in non-high-risk disease, with a chemotherapy-free regimen of all-trans retinoic acid (ATRA) and arsenic trioxide (ATO) once the diagnosis is confirmed; high-risk presentations commonly add cytoreduction (1). That confirmation rests on genetics. Interphase FISH for the PML::RARA fusion, karyotyping of a metaphase spread, and reverse-transcription PCR for the fusion transcript are the tests that separate APL from other AML subtypes. The Sanz risk group used to guide induction therapy is assigned from the presenting white-cell and platelet counts alone. Molecular and cytogenetic testing remains essential for establishing the diagnosis and for guiding overall management, and it is on that testing, not on the risk score, that the decision to treat as APL rests. A Wright–Giemsa bone marrow smear is read alongside these tests, and its hypergranular promyelocytes are often what first raises clinical suspicion. Morphology can point toward an underlying rearrangement without identifying it, so the smear supports the genetic tests rather than substituting for them. In most laboratories, the smear, the FISH slide, and the karyotype preparation are examined separately, typically by different technologists and often on different timelines, although practice varies between institutions.

This separation is not only a matter of convenience. FISH and karyotype preparations use fixation, hybridization, or banding protocols that differ from a routine smear, and the three examinations are reported independently even though all three describe the same patient.

### 1.2 Residual non-leukemic marrow composition

Leukemia is not only a disease of malignant cells. The surrounding marrow retains a mixture of normal lymphocytes, monocytes, and maturing myeloid and erythroid precursors, and their relative abundance changes as leukemic infiltration replaces normal hematopoiesis. This is tracked clinically, in a coarse way, through the blast percentage on a differential count, but a differential count collapses a wide range of normal lineages into a small number of categories and treats the malignant clone as the only quantity of interest. How much of the marrow’s residual non-leukemic tissue remains, as distinct from how much is occupied by leukemic cells, is not routinely reported.

In APL, host immune function is known to be perturbed by treatment itself: ATO delays the reconstitution of circulating natural killer cells for well over a year after single-agent therapy, even as it sensitizes leukemic promyelocytes to natural killer cell killing (2). Those observations concern properties of the marrow environment that the readout developed here does not measure: a Wright–Giemsa differential resolves morphologic cell classes, not immune phenotypes, activation states, or the cytokine milieu. A per-patient summary of residual non-leukemic marrow composition at diagnosis, read from the same smear used for morphologic diagnosis, has not to our knowledge been attempted in this disease.

### 1.3 Image-based cytogenetics and differentiation syndrome

Reading a fusion pattern from an interphase FISH image, or a structural abnormality from a metaphase spread, is currently a task for a cytogeneticist counting fluorescent signals or arranging chromosomes by eye. Automated spot detection exists: U-FISH counts and localizes fluorescent signals across several fusion and break-apart probes, including the PML::RARA dual-fusion probe used in APL, but it stops at counting signals and does not classify the fusion-pattern class that carries clinical weight (3). On the karyotype side, a vision transformer pipeline trained on thousands of patients reads chromosomal abnormalities directly from raw metaphase images at an area under the curve (AUC) of 0.94 for del(5q) and t(9;22) and can flag rare aberrations it was never trained on (4), but this pipeline, like others in the same line of work, targets aberrations outside APL. Cytomorphology has its own precedent: deep learning models distinguish APL from other AML on a bone marrow or peripheral smear at a level comparable to practicing hematopathologists (5, 6). We did not identify a published model trained on two or more of these image types in the same patients with APL (**Supplementary Table 7**), and a recent review of artificial intelligence applications in APL reported no study that works with FISH or karyotype images in this disease, and none that predicts differentiation syndrome from any data type (7).

Differentiation syndrome (DS) is the treatment-related complication that motivates asking whether the genetic complexity axis relates to it. The syndrome follows leukocyte activation and capillary leak as ATO and ATRA drive promyelocyte differentiation, and it remains a cause of early death despite prophylactic corticosteroids. Whether structurally complex or atypical cytogenetics raises DS risk has been posed as a clinical question, but the two studies that tested cytogenetic or molecular complexity against DS occurrence directly reported no significant association; the syndrome instead tracked peak white-cell count, coagulopathy, and hypoalbuminemia (8, 9). We therefore treat any relationship between image-derived genetic features and DS as an exploratory question this cohort allows us to probe, not a premise we assume.

### 1.4 Contributions

**Figure 1:**
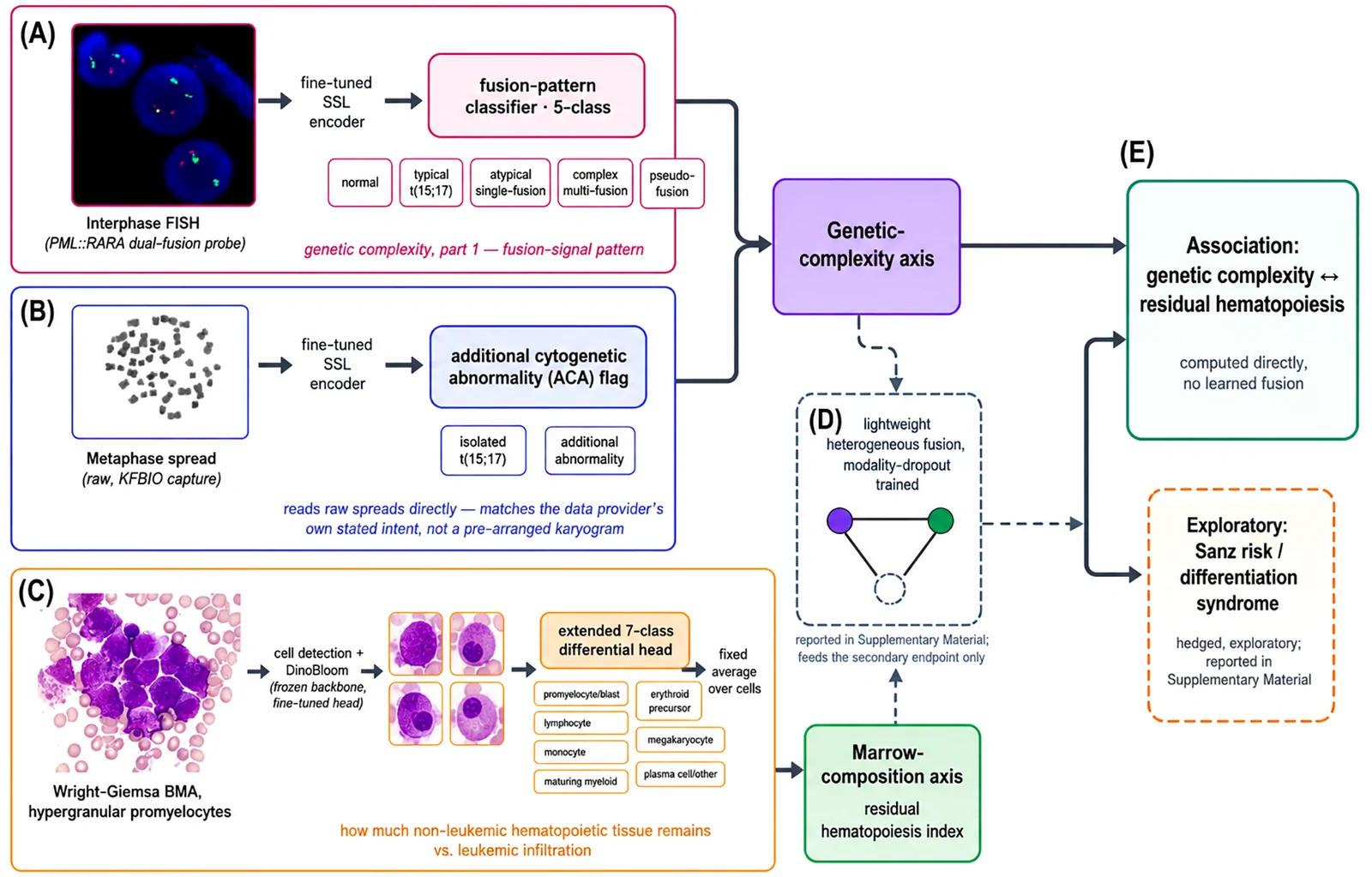
Framework overview. **(A)** The FISH branch classifies the PML::RARA fusion signal pattern into five classes with a fine-tuned self-supervised encoder. **(B)** The karyotype branch flags any additional cytogenetic abnormality beyond the t(15;17) on raw metaphase spreads. **(A)** and **(B)** form the genetic complexity axis. **(C)** The smear branch detects cells, embeds them with a frozen DinoBloom backbone and a directly supervised per-cell head, and averages over cells to form the residual hematopoiesis index of the marrow composition axis. **(D)** A lightweight three-node fusion module supports the secondary endpoint only and is reported in the Supplementary Material. **(E)** The primary result is the association between genetic complexity and residual marrow hematopoiesis, computed directly from the branch outputs without the fusion module.

This study contributes three purpose-built readers, one descriptive association, and one null. First, a classifier assigns an interphase FISH image to a fusion-pattern class rather than only counting its signals. Second, a reader flags any additional cytogenetic abnormality (ACA) beyond the t(15;17) on raw, unarranged metaphase spreads, the form in which the data underlying this study were collected. Third, a marrow cytomorphology pipeline is extended from promyelocyte detection to a seven-class differential, from which a residual hematopoiesis index, the estimated fraction of non-leukemic nucleated marrow cells, is computed. The association between image-derived genetic complexity and this index is the primary finding, reported across 700 patients together with the adjustments and substitutions applied to it. An exploratory test of the same readouts against the Sanz risk group and DS occurrence is reported as a null. The three readers share a cohort and a label taxonomy. The primary result is computed from their outputs by a fixed formula with no fitted parameters, so it rests on the branch outputs themselves. **Figure 1** summarizes the resulting framework.

## 2 Related work

### 2.1 Automated analysis of bone marrow cytomorphology

Deep learning applied to bone marrow or peripheral blood smears distinguishes APL from other AML subtypes with an accuracy that approaches expert hematopathologists. A multiple-instance learning (MIL) model trained without cell-level annotation separates the two conditions in blood films and bone marrow aspirates with an AUC of 0.94 and 0.99, respectively (10), and a single-cell convolutional network trained on peripheral smears alone matched or exceeded 10 hematopathologists reading the same slides (5). A related pipeline combines cell segmentation with three binary classifiers for myeloblasts, promyelocytes, and Auer rods to separate APL from other AML (6). Beyond diagnosis, a genotype prediction pipeline built on single-cell images from Pappenheim-stained smears predicts clinically actionable mutations such as NPM1 and FLT3-ITD directly from morphology, without reference to a genetic test (11). Most of these pipelines build per-cell representations from a convolutional network trained from scratch or on natural images; the cytomorphology branch of this study instead uses DinoBloom, a self-supervised Vision Transformer (ViT) trained on more than 380,000 white-cell images pooled across 13 public hematology datasets, whose embeddings generalize across smear preparations and staining batches (12). The smear branch of this study follows the same pattern: a shared self-supervised encoder paired with a small, task-specific head. DinoBloom itself was evaluated on a multiclass bone marrow cell-typing task (12), and automated marrow differentials have been built with other encoders. These pipelines report cell types. None summarizes the residual non-leukemic composition as a single per-patient readout, and none relates such a readout, in the same patients, to image-derived genetic features in APL.

### 2.2 Automated analysis of interphase FISH images

Interphase FISH scoring is, in most laboratories, a manual task: a cytogeneticist counts red, green, and fusion signals across dozens of nuclei and reports the proportion of nuclei with an abnormal pattern. Automating that count is not new outside leukemia. In HER2-amplified breast cancer, a RetinaNet-based pipeline detects interphase nuclei and classifies HER2 and centromere-17 signals to assign an amplification category that matches manual scoring across several hundred cases (13), and a more recent model applies the same signal-counting logic to a five-group amplification score defined by current clinical guidelines and reaches 85% classification accuracy against manual review (14). U-FISH generalizes this counting step across probe types rather than one cancer’s amplification assay: it is a spot detector trained across seven imaging sources and more than 1.6 million annotated signals, and it has been demonstrated directly on PML::RARA dual-fusion images among other probe types used in leukemia (3). What all three pipelines share is a stopping point at the signal count or a fixed amplification category; none classifies the qualitatively different fusion-pattern class, a typical balanced fusion against a single-fusion or a multifusion pattern, that a cytogeneticist distinguishes when reading a PML::RARA study and that flags an atypical or cryptic rearrangement.

### 2.3 Automated analysis of karyotype and metaphase images

A line of work automates the earlier steps of karyotyping: Varifocal-Net classifies each chromosome’s type and polarity from a cropped image at an accuracy of 99.2% per case (15), DeepACEv2 automates chromosome enumeration (16), and ChromTR detects and classifies chromosomes directly in raw, unseparated metaphase images with a deformable transformer architecture, at an average precision of 92.56% (17). A separate line targets structural abnormality directly: a vision transformer pipeline trained on more than 6,000 patients reads del(5q) and t(9;22) from raw metaphase images at an AUC of 0.94 and can flag aberrations it has never seen labeled (4). Each of these pipelines was built and evaluated on chromosome types or aberrations outside APL; none targets t(15;17) or the additional aberrations, such as trisomy 8 or an isochromosome 17, that a karyotype in this disease is read for.

### 2.4 Multimodal fusion across heterogeneous and sparse imaging modalities

Fusing imaging modalities that differ in how many images exist per patient is an open methodological problem beyond leukemia. Most architectures in computational pathology pair many patches from a whole-slide image with a fixed-length vector of genomic or clinical features, joined by cross-attention, and the prototype-based alternatives that replace that step still fuse one many-instance modality with one fixed-length one (18–20). Robustness to a modality missing at test time has been addressed separately, through shared latent spaces and gradient decoupling (21, 22), and two pipelines address the specific mismatch of a sparse modality against a many-instance one (23, 24). A bone marrow smear yields dozens of cell images per patient, while an interphase FISH or metaphase karyotype examination yields a handful, so a fusion module sized to a cohort like this one should carry few trainable parameters and should not require every patient to contribute every modality.

### 2.5 Differentiation syndrome and its clinical risk factors

Differentiation syndrome was characterized in a cohort of 739 patients treated with ATRA and idarubicin, where the variables associated with its severe form included a white-cell count above 5 × 10^9^ per liter, abnormal creatinine, an FLT3-ITD mutation, the microgranular morphologic subtype, and the short PMLRARA isoform (25). A retrospective cohort of 316 patients treated between 2014 and 2024 confirmed peak white-cell count, hypoalbuminemia, and the absence of prophylactic corticosteroids as independent predictors, and found no association between the syndrome and any cytogenetic or molecular feature it tested (8). A separate cohort of 171 patients found the same absence of association between additional cytogenetic abnormalities and DS incidence, although the same abnormalities predicted worse event-free survival over a 5-year follow-up (9), a relapse association echoed in an arsenic-trioxide-treated cohort in which a complex, but not merely additional, karyotype predicted inferior event-free survival (26). Karyotype complexity, in other words, has documented value for relapse; it has not been shown to predict DS. A recent review of artificial intelligence applications in this disease found no model that predicts the syndrome from any of these variables, image-derived or otherwise (7).

## 3 Materials and methods

### 3.1 Cohort, data provenance, and label taxonomies

**Figure 2:**
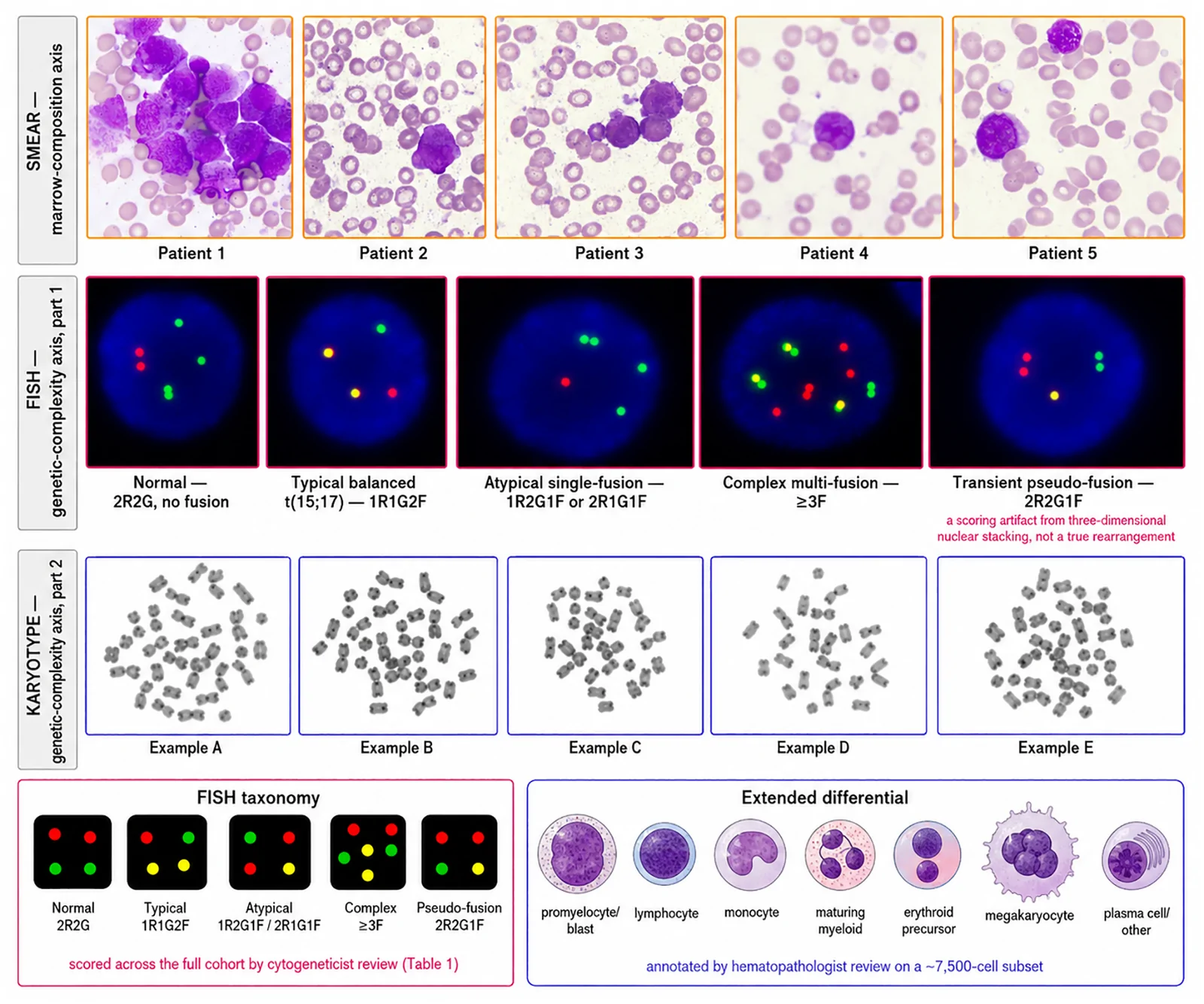
Data and label taxonomy. Cropped fields from the three modalities of the linked cohort: bone marrow smear (marrow composition axis), interphase FISH, and metaphase karyotype (genetic complexity axis). The FISH row shows one field of each of the five classes of **Table 2**, captioned with the class assigned by the two cytogeneticists’ consensus, with the atypical, complex, and pseudo-fusion examples given the most space. The smear and karyotype rows carry no per-field class label, since none was assigned for those modalities. The taxonomy legends below describe the cohort-wide annotation schema applied by cytogeneticist review and by hematopathologist review (seven-class differential; **Table 3**).

We assembled a retrospective cohort of patients with newly diagnosed PML::RARA-positive APL at The First Affiliated Hospital of Ningbo University between January 2014 and December 2025. A patient entered the study with a bone marrow aspirate smear, an interphase FISH study for the PML::RARA fusion, and a metaphase karyotype, all obtained at diagnosis and linked to the same patient record. Requiring all three examinations may exclude patients with failed metaphase growth or an incomplete workup, a selection effect addressed in Section 5. The cohort holds 700 patients meeting this criterion. Because these confirmed cases are PML::RARA-positive by inclusion and so contribute no normal pattern FISH examples, we added 150 FISH studies from patients worked up for suspected disease at the same laboratory and over the same period who tested PML::RARA-negative, so that the fusion-pattern classifier has real examples of its normal class rather than none. The Sanz risk group and DS status were available for 650 of the 700 confirmed patients; the remaining 50 had an incomplete clinical record and are excluded from the analyses that use these fields, a missingness we do not assume to be at random. **Table 1** and **Figure 2** summarize the cohort, its subcohorts, and its clinical characteristics.

**Table 1:** Cohort, subcohort, and clinical characteristics.

Item	Value
Patients, full cohort (smear + FISH + karyotype linked)	700
Patients excluded (incomplete workup/no evaluable karyotype)	200 of 900 (120/80)
FISH-negative controls (normal class training)	150
Clinical annotation subcohort (Sanz risk, DS status)	650 of 700 (92.9%)
Cytogeneticist-scored FISH-pattern labels	850 (700 + 150 controls)
Hematopathologist-annotated differential cell set	7,500 cells/60 patients
Median age, years (IQR), of 700	44 (33 to 56)
Male, of 700	343 (49.0%)
Sanz risk: low/intermediate/high, of 650	156 (24.0%)/299 (46.0%)/195 (30.0%)
White-cell count at presentation, ×10^9^/L, median (IQR), of 650	3.8 (1.6 to 14.2)
Platelet count at presentation, ×10^9^/L, median (IQR), of 650	24 (14 to 41)
Morphologic subtype: hypergranular/microgranular, of 700	588 (84.0%)/112 (16.0%)
Accession period: 2014–2019/2020–2025, of 700	289 (41.3%)/411 (58.7%)
FISH pattern: normal/typical/atypical/complex/pseudo-fusion, of 850 images	145 (17.1%)/627 (73.8%)/43 (5.1%)/26 (3.1%)/9 (1.1%)
Karyotype: isolated t(15;17)/additional abnormality, of 700	476 (68.0%)/224 (32.0%)
Metaphase spreads examined per patient, median (IQR), of 700	20 (15 to 25)
Scorable nuclei per FISH image, median (IQR), of 850	58 (44 to 76)
Differentiation syndrome occurrence, of 650	189 (29.1%)
PML::RARA isoform available; bcr1/bcr2/bcr3	402 of 700; 233/24/145
FLT3-ITD tested; positive	178 of 700; 61 (34.3%)
Diagnostic flow cytometry retrievable	118 of 700
Slides re-scanned for the reproducibility check	80 of 700
Data lock	31 March 2026

Counts are given alongside every percentage. Denominators differ by row and are stated in the item column.

The data were locked on 31 March 2026. Differentiation syndrome is an induction-phase event assessed within the first 30 days of therapy, and the last patients accrued in December 2025 had completed induction by the end of February 2026, so ascertainment is complete for the most recent accessions.

Presenting white-cell and platelet counts, the two fields from which the Sanz risk group is assigned were retrieved for the 650 patients with a complete clinical record and are reported in **Table 1**. Morphologic subtype, PML::RARA isoform, and FLT3-ITD status were recorded where available: subtype for all 700 patients, isoform for 402, and FLT3-ITD for the 178 patients in whom it was tested. Routine diagnostic multiparameter flow cytometry, performed at diagnosis on the same aspirate, was retrievable for 118 patients; the panel used CD45 and side scatter for gating together with CD3, CD19, CD34, CD117, CD13, CD33, and myeloperoxidase, and we derived from it the fraction of nucleated events falling outside the abnormal promyelocyte and blast gate. That fraction is the external comparator for the residual hematopoiesis index defined in Section 3.2. **Supplementary Table 8** gives the panel and the gating definition.

Of 900 patients with confirmed PML::RARA-positive APL over the study period, 120 lacked at least one of the three linked examinations and a further 80 had no evaluable metaphase karyotype, leaving the 700 analyzed here. A scanned smear was retrievable for 132 of the 200 excluded patients, which allows the excluded patients to be characterized; that comparison is reported in Section 4.6.

Two label taxonomies structure the genetic complexity and marrow composition axes described in Sections 3.2 to 3.4. The FISH fusion-pattern classifier is trained toward five classes, adapted from the interphase FISH signal-pattern scheme used in routine cytogenetic reporting for PML::RARA (**Table 2**): a normal pattern with two red and two green signals and no fusion; a typical balanced t(15;17) pattern with one red, one green, and two fusion signals; an atypical single-fusion pattern with one fusion signal and an unequal red-green count, which can arise from an insertion, a deletion, or a variant rearrangement; a pattern with three or more fusion signals; and a transient pseudo-fusion pattern arising from three-dimensional nuclear stacking rather than a true rearrangement, which is a scoring artifact rather than a patient genetic class. Two cytogeneticists independently scored all 850 FISH images (700 positive cases and 150 negative controls) against this taxonomy, agreeing on the five-class label before discussion in 88% of images (Cohen’s *κ* = 0.79); the remaining disagreements, concentrated in the rare atypical and pseudo-fusion classes, were resolved by consensus. The resulting class counts are 145 normal, 627 typical, 43 atypical, 26 complex, and 9 pseudo-fusion (**Table 1**). Three of these classes therefore rest on tens of images, which bounds what the per-class performance estimates can support and is restated in Section 5.

**Table 2:** FISH fusion signal pattern taxonomy.

Class	Signal pattern	Interpretation
Normal	2R2G, no fusion	No PML::RARA fusion detected
Typical balanced t(15;17)	1R1G2F	Balanced reciprocal fusion pattern
Atypical single fusion	1R2G1F or 2R1G1F	Single-fusion pattern; insertion, deletion, or variant rearrangement
Complex multifusion	≥3F	Three or more fusion signals
Transient pseudo-fusion	2R2G1F	Nuclear stacking artifact, not a true rearrangement

The extended marrow differential (**Table 3**) enlarges the per-cell classification target from a binary leukemic-versus-normal call to seven classes: leukemic promyelocyte or blast, lymphocyte, monocyte, maturing myeloid cell, erythroid precursor, megakaryocyte, and a residual plasma cell or other category. This taxonomy, not the standard clinical differential, is what the smear branch’s per-cell head is trained to predict, and it is the direct input to the residual hematopoiesis index of Section 3.2. A hematopathologist annotated 7,500 cells across 60 patients to supervise this head, following a written protocol with defined handling of ambiguous and degenerate cells; the per-class counts run 3,100 promyelocyte or blast, 1,450 lymphocyte, 520 monocyte, 1,180 maturing myeloid, 890 erythroid precursor, 180 megakaryocyte, and 180 plasma cell or other and are listed with the reference standard for every supervised readout in **Table A1** in **the Appendix**. The 60 patients were drawn at random from the 700, stratified on the Sanz risk group and accession year, without enrichment for rare cell classes; the megakaryocyte and residual classes are therefore supervised from few cells, which is visible in their per-class metrics below. Because a single reader annotated the training cells, the label set carries that reader’s convention; a second hematopathologist re-scored 750 of the cells, agreeing on the seven-class label in 84% (Cohen’s *κ* = 0.78), and this interreader variability in the marrow differential bounds the residual hematopoiesis index, a limitation addressed in Section 5.

**Table 3:** Extended marrow differential taxonomy.

Class	Description
Promyelocyte/blast	Leukemic cell
Lymphocyte	Non-leukemic lymphoid lineage
Monocyte	Non-leukemic monocytic lineage
Maturing myeloid cell	Myelocyte through segmented neutrophil
Erythroid precursor	Non-leukemic erythroid lineage
Megakaryocyte	Non-leukemic megakaryocytic lineage
Plasma cell/other	Residual category

Treating the promyelocyte and blast class as leukemic is a definitional assumption, stated here rather than left implicit. It holds for a cohort of untreated, PML::RARA-positive patients at diagnosis, where normal promyelocytes are a negligible share of the differential. It would not hold in a regenerating or post-induction marrow, where a granulocytic left shift produces normal promyelocytes in quantity, and this is a barrier to any longitudinal application of the index.

A cohort of this size gives the primary genetic complexity/residual hematopoiesis association reasonable power; the secondary Sanz risk and DS analyses, run on the 650-patient clinical-annotation subcohort, are described as such throughout rather than presented as full-cohort findings, and no analysis here claims validity beyond a single-center, retrospective population.

### 3.2 Smear branch—extended marrow differential

Each patient’s diagnostic smear comprises one or more digitized 40× Wright–Giemsa fields, read from cellular, non-crushed areas away from the smear edge on a single slide scanner at 0.25 microns per pixel; a median of 8 fields (interquartile range 5 to 12) and 237 nucleated cells (interquartile range 176 to 314) passed the fixed focus and staining quality-control criteria per patient. Slides were scanned and fields selected before the cytogenetic report was issued, so field selection was blinded to FISH and karyotype status throughout the cohort. **Supplementary Table 4** gives the scanner, stain, probe, microscope, and banding details for all three modalities. Nucleated cells are located and segmented from each field with Cellpose (27), a generalist cell-segmentation network; each detected cell is cropped to a fixed-size patch centered on its nucleus. Because detection error propagates into the composition endpoint, the segmentation was checked against 40 held-out fields with manually marked cells, reaching a detection F1 of 0.94 (precision 0.95, recall 0.93) before use. Megakaryocytes are too large for an ordinary nucleus-centered crop, so they are detected with a size-adapted crop. They form their own class rather than being merged into the residual category, and they are counted in the denominator of Equation 2 and in the class sum of Equation 3 like any other class. Because megakaryocytes concentrate near spicules and the feathered edge while the protocol reads fields away from the edge, this class is likely undersampled; we report the index recomputed without it as a check. A frozen DinoBloom-B encoder, a ViT operating on 16×16 patches with a 768-dimensional output (12), maps each cropped cell to an embedding $e_{i}\in {\mathbb{R}}^{768}$; the encoder’s weights are not updated at any stage.

A single linear layer,

(1) $$p_{i}=\text{softmax}(We_{i}+b),\;\;W\in {\mathbb{R}}^{7\times 768},\;b\in {\mathbb{R}}^{7}$$

maps each cell embedding to a probability vector *p_i_* ∈ Δ^6^ over the seven classes of **Table 3**, with class 1 denoting leukemic promyelocyte or blast. This linear layer is the only trained component of the smear branch: its weights are fit by supervised cross-entropy on the 7,500 hematopathologist-labeled cells from 60 patients (**Table 1**). Training and validation follow the same patient-stratified five-fold cross-validation with five repeats used throughout this study (Section 3.7), so that no patient contributes cells to both a training and a validation fold within any repeat. Real cell-level labels exist for this task, unlike the patient-level cytogenetic labels this lab’s earlier cytomorphology models were trained against, so the head is supervised directly rather than through attention-based MIL: no bag-level aggregation is fit to substitute for missing instance labels, because none are missing here.

The fitted linear layer is then applied, without further training, to every detected cell in all 700 patients of the cohort. For patient *j* with *N_j_* detected cells, the marrow composition is the unweighted mean of the per-cell class probabilities,.

(2) $$\pi_{j}=\frac{1}{N_{j}}\sum_{i=1}^{N_{j}}p_{i}\in {\text{Δ}}^{6},$$

so that *π_j,k_* is the estimated fraction of patient *j*’s marrow cells belonging to class *k*. Equation 2 is a fixed average with no parameters of its own; the composition vector is fully determined by Equation 1 applied cell by cell, not by a separately trained aggregation step.

The residual hematopoiesis index is the fraction of a patient’s classified cells that fall outside the leukemic class,

(3) $$R_{j}=1-\pi_{j,1}=\sum_{k=2}^{7}\pi_{j,k}\in \left[0,1\right],$$

so *R_j_* = 0 describes a marrow read as entirely leukemic promyelocytes or blasts and *R_j_* = 1 describes a marrow with no cells classified as leukemic; a higher *R_j_* therefore indicates a larger non-leukemic fraction. *R_j_* is a model-estimated cell fraction, and its interpretation rests on the per-cell classifier being adequately calibrated, a limitation addressed in Section 5. It is the smear branch’s scalar contribution to the marrow composition axis; the full seven-class vector *π_j_* is retained for the composition results of Section 4.4.

*R_j_* is a per-patient summary of the non-promyelocyte fraction of a Wright–Giemsa aspirate differential, computed by a classifier over cell images, and serves as a morphology-derived surrogate for residual non-leukemic marrow composition. Its resolution is that of the stain: morphologic cell classes and their relative proportions. Immune phenotype, activation and exhaustion state, the cytokine milieu, and spatial organization are addressed by immunophenotyping, cytokine assay, and spatial profiling and lie outside the measurements this study holds.

Two checks bound the reliability of the quantity. First, diagnostic flow cytometry was retrievable for 118 patients (Section 3.1), and the correlation between *R_j_* and the flow-derived non-blast fraction is reported in Section 4.4. The two measurements count different denominators, and hemodilution of an aspirate affects them differently, so the comparison characterizes agreement rather than providing an independent reference standard. Second, 80 slides drawn at random from the 700 were re-scanned, and their fields were independently re-selected by a second operator who was blinded to FISH and karyotype status and to the first operator’s field choices. We report the intraclass correlation of *R_j_* across that pair, which carries both the scanning and the field selection contribution to variability. We also propagate the binomial sampling error of a finite differential into a per-patient interval for *R_j_* and report an interval on the cohort median.

### 3.3 FISH branch—fusion-pattern classification

Each of the 850 interphase FISH images (**Table 1**: 700 from confirmed patients, 150 negative controls) is resized to 224 × 224 pixels and normalized per color channel; no cropping is applied, since the informative signal, the red PML, green RARA, and yellow fusion puncta, is distributed across the field rather than confined to a fixed region. Random rotation and flipping are applied during training, since a nucleus has no canonical orientation. Each patient contributes exactly one FISH image and receives exactly one label from **Table 2**, so the FISH branch is an ordinary per-image classification task and does not require the cell-to-patient averaging the smear branch applies.

Each patient’s FISH study was captured as a set of fields, from which one field per patient was selected for analysis by a cytogenetic technologist who was not one of the two scoring cytogeneticists, on the fixed criterion of the largest number of in-focus, non-overlapping nuclei, blinded to the karyotype report and to all clinical data but not to the FISH report itself. The selected field carries a median of 58 scorable nuclei (interquartile range 44 to 76, range 21 to 142). A field chosen for signal clarity may under-represent the overlapping nuclei from which pseudo-fusion artifacts arise, so the number of scorable nuclei is tested against the branch output in Section 4.6.

Assigning one pattern class to a whole image, and through it to a patient, departs from how the assay is read clinically. In routine practice, an interphase FISH study is scored across roughly 200 nuclei and reported as the proportion showing each pattern, and pseudo-fusion from three-dimensional stacking is a per-nucleus artifact whose expected rate is what a laboratory cutoff is set against. The image-level formulation follows the form in which these data were archived, one representative field per patient. A nucleus-level model producing a per-patient distribution over patterns would correspond more closely to clinical reporting and is considered in Section 5.

No FISH-specific foundation model exists, unlike DinoBloom for cytomorphology, so the FISH branch fine-tunes a general-purpose self-supervised Vision Transformer, DINOv2 ViT-S/14 (28), initialized from its publicly released weights. Every encoder weight is updated during training, in contrast to the smear branch’s frozen backbone, because adapting the encoder’s features to fluorescence images with no in-domain pretraining counterpart is the point of this branch, and the 850-image cytogeneticist-scored set is large enough to support end-to-end fine-tuning rather than the head-only training used for the smear branch’s 60-patient annotation set.

The encoder’s class token $z_{j}=f_{\phi }(x_{j})\in {\mathbb{R}}^{384}$ for image *x_j_* feeds a linear head,

(4) $$q_{j}=\text{softmax}(Vz_{j}+c),\;\;V\in {\mathbb{R}}^{5\times 384},\;c\in {\mathbb{R}}^{5}$$

over the five classes in **Table 2** in the order listed there: normal, typical balanced fusion, atypical single fusion, complex multifusion, and transient pseudo-fusion. Because these classes are unevenly represented (**Table 1**: 17%, 74%, 5%, 3%, and 1% of the 850 images), the encoder and head are trained jointly by minimizing a class-balanced cross-entropy loss,

(5) $$ℒ=-\frac{1}{M}\sum_{j=1}^{M}w_{y_{j}}\log\;q_{j,y_{j}},\;w_{k}=\frac{M}{5n_{k}},$$

where *y_j_* is image *j*’s true class, *M* is the number of training images in a given fold, and *n_k_* is the number of training images of class *k* in that fold, so the loss weights the five classes equally in expectation rather than in proportion to their frequency.

The encoder and head are evaluated by five-fold cross-validation with five repeats, stratified on class label. Because each patient contributes a single image, this is equivalent to the patient-stratified scheme used for the other two branches (Section 3.7) and carries no risk of one patient’s data appearing in both a training and a validation fold.

### 3.4 Karyotype branch—additional abnormality flag

The ACA status is defined at the patient level, within the 700 confirmed patients only: 476 (68%) show the defining t(15;17) translocation alone, and 224 (32%) carry at least one additional cytogenetic abnormality alongside it, established from each patient’s full cytogeneticist karyotype report (**Table 1**). An ACA may be a single numerical or structural change, such as trisomy 8, and does not by itself constitute a complex karyotype in any standard sense; the flag reads the presence of any additional abnormality, not karyotype complexity. This distinction does not apply to the 150 FISH-negative controls, which are not part of the karyotype branch. Each patient’s karyotype study yields a variable number of raw, unarranged metaphase spread images, one per examined metaphase cell; an ACA need not be visible in every spread from a given patient, since not every metaphase captured during a study shows every clone present.

Each spread image is thresholded to separate chromosome material from the blank slide background, cropped to the bounding region of that material with a fixed margin, and resized to 224 × 224 pixels, so the classifier sees the scattered chromosomes at a consistent scale rather than a field dominated by empty background. As with the FISH branch, no pre-arranged karyogram is used at any stage: the classifier reads the raw, unsplit spread directly. A DINOv2 ViT-S/14 encoder, the same architecture family as the FISH branch but a separately initialized and fine-tuned instance, maps each spread image *x_j,l_* (patient *j*, spread *l*) to a class token $z_{j,l}\in {\mathbb{R}}^{384}$; CHROMA (29) was considered but not used here, since it is pretrained on cropped individual chromosomes rather than whole spreads, and adopting it would require an unspecified chromosome segmentation step this design avoids by reading the whole field directly.

A linear layer with a single output unit,

(6) $$r_{j,l}=\sigma (v^{⊤}z_{j,l}+c_{0}),\;\;v\in {\mathbb{R}}^{384},\;c_{0}\in \mathbb{R}$$

gives the estimated probability that spread *l* shows an additional abnormality beyond t(15;17). During training, every one of a patient’s spreads inherits that patient’s composite patient ACA label *y_j_* ∈{0,1}, since no per-spread expert relabeling exists; encoder and head are fit jointly with a class-balanced binary cross-entropy,

(7) $$ℒ=-\frac{1}{M}\sum_{j,l}[w_{1}\;y_{j}\log\;r_{j,l}+w_{0}\;\left(1-y_{j}\right)\log\;(1-r_{j,l})],\;w_{1}=\frac{M}{2n_{1}},\;w_{0}=\frac{M}{2n_{0}},$$

where *M* is the number of training spreads in a fold and *n*_0_, *n*_1_ are the number of those spreads inheriting each label.

**Figure 3:**
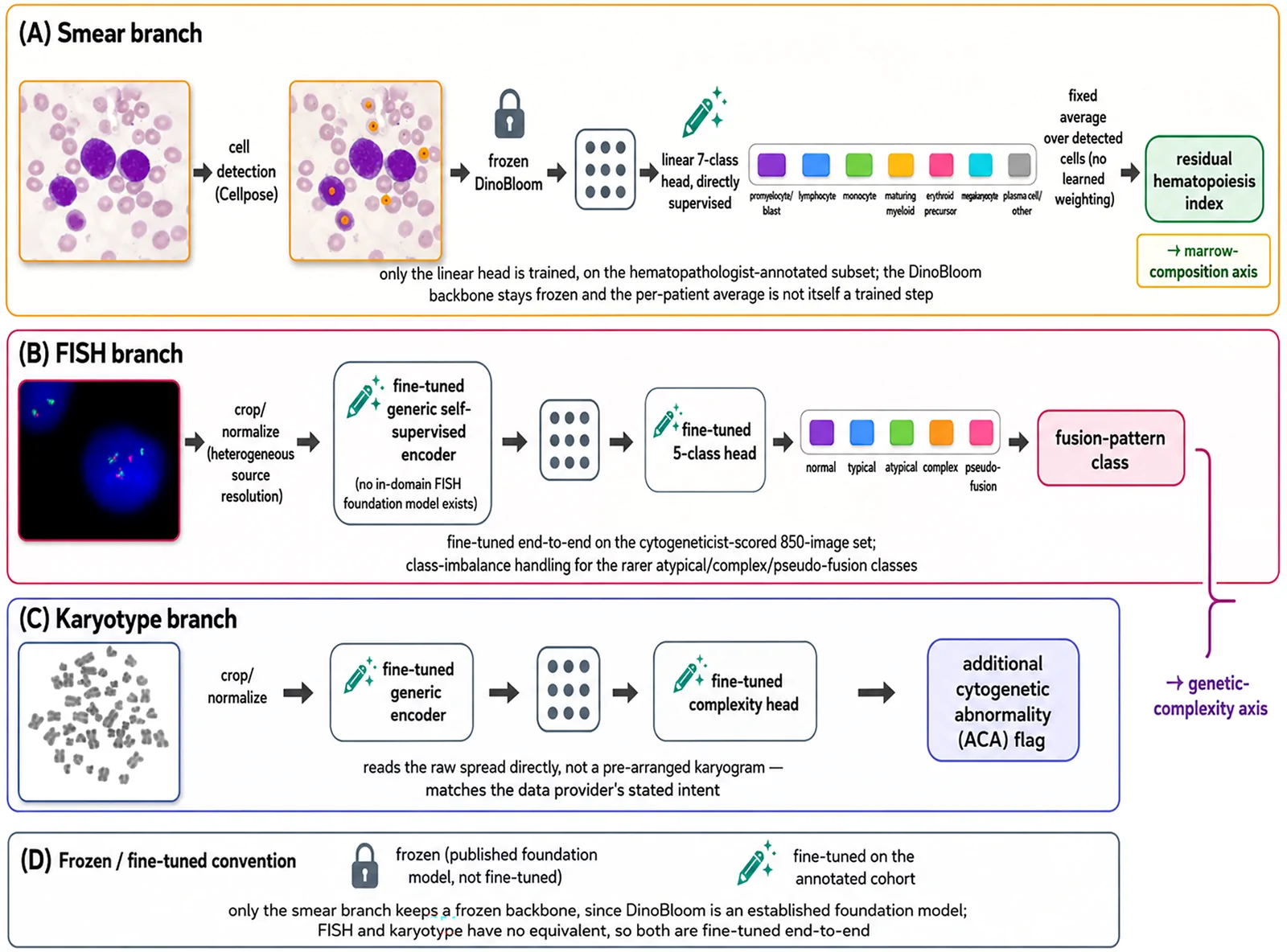
Per-modality branch architecture. **(A)** Smear branch: Cellpose cell detection, a frozen DinoBloom backbone, a directly supervised linear seven-class head, and a fixed average over detected cells yielding the residual hematopoiesis index. **(B)** FISH branch and **(C)** karyotype branch: a generic self-supervised encoder and a classification head, both fine-tuned end-to-end, since no in-domain foundation model exists for either. **(D)** The frozen-versus-fine-tuned convention marked throughout: only the smear branch keeps a frozen backbone.

At inference, patient *j*’s ACA score is the mean of the spread-level probabilities of Equation 6,

(8) $$\rho_{j}=\frac{1}{L_{j}}\sum_{l=1}^{L_{j}}r_{j,l},$$

where *L_j_* is the number of spreads examined for patient *j*; a patient is flagged as ACA-positive when *ρ_j_* exceeds a threshold fixed at 0.5. The metaphase yield *L_j_* has a median of 20 spreads (interquartile range 15 to 25, range 8 to 34). Yield is the variable on the ascertainment pathway that could link marrow composition to detected complexity, since a marrow less densely packed with leukemic cells may culture better and so present more spreads in which an additional abnormality can be seen. We therefore treat *L_j_* as a recorded quantity and test both axes against it in Section 4.6. As in the smear branch, Equation 8 is a fixed average with no parameters of its own, so the patient-level flag is fully determined by the spread-level classifier. Because every spread inherits the patient’s single report label, the per-spread output *r_j,l_* is a weak label score rather than a validated spread-level lesion detection, and the 0.5 threshold is a reporting convention rather than a calibrated operating point; the ACA reader is therefore evaluated at the patient level only. Validation follows the patient-stratified five-fold, five-repeat scheme used throughout this study, so that no patient’s spreads split across a training and a validation fold. **Figure 3** summarizes the construction of all three branches side by side, with the frozen and fine-tuned components of each marked explicitly.

### 3.5 Fusion module for the secondary endpoint

The fusion module produces a single learned representation per patient, *z_j_*, from the three branch outputs, and it feeds the secondary, exploratory endpoint alone. The primary endpoint does not use *z_j_*: it is computed directly from the branch outputs by a formula with no learned parameters, as set out in Section 3.6.

Its architecture, its modality-dropout training, the ablation, and the Shapley decomposition are set out in the **Supplementary Material** together with **Supplementary Figure 1**. The module operates on the 700 confirmed patients; the karyotype ACA flag is undefined for the 150 FISH-negative controls, which therefore do not enter it, and no quantity downstream of it reaches the primary result.

### 3.6 Endpoints

#### 3.6.1 Primary endpoint

A genetic complexity score is computed directly from the FISH and karyotype branch outputs, with no learned parameters. The FISH branch’s atypicality score is the probability mass its classifier places on the atypical, complex, or pseudo-fusion classes rather than normal or typical (**Table 2**),

(9) $$F_{j}=q_{j,3}+q_{j,4}+q_{j,5}=1-q_{j,1}-q_{j,2}$$

using the class order of Equation 4. The genetic complexity score combines this with the karyotype ACA score of Equation 8 in an equally weighted average,

(10) $$G_{j}=\frac{1}{2}F_{j}+\frac{1}{2}\rho_{j}\in [0,1]$$

The primary result of this study is the Spearman rank correlation between *G_j_* and the residual hematopoiesis index *R_j_* (Equation 3), computed across the 700 confirmed patients and reported with a bootstrap confidence interval (CI) over patients and a *p*-value. Equations 9 and 10 add no learned parameters of their own, so the correlation does not depend on the fusion module of Section 3.5; it is a fixed function of the branch outputs of Equations 4 and 8. Those outputs are themselves model predictions, however, so *G_j_* and *R_j_* inherit the branches’ error and calibration; for the primary analysis, each patient’s *G_j_* and *R_j_* are therefore taken from branch models fit on folds that excluded that patient, so no patient’s value is produced by a model trained on it.

Three properties of Equation 10 are modeling choices rather than definitions, and we state them as such. The equal weighting of *F_j_* and *ρ_j_* is a reporting convention; the two components are on the same nominal scale but are not calibrated against each other, and no data were used to fit the weights. The mapping from fusion pattern to complexity is also a choice: an atypical single-fusion pattern arising from a cryptic insertion is not, in any genomic sense, more complex than a typical balanced fusion, and *F_j_* tracks departure from the expected balanced pattern. The pseudo-fusion class contributes positively to *G_j_* even though it is a scoring artifact rather than a patient property. We therefore report the composite alongside its components rather than in place of them, and we report the association recomputed at unequal weightings and with the pseudo-fusion mass removed.

Because *G_j_* is heavily concentrated, with 68% of patients carrying the isolated translocation and 74% of images a typical fusion pattern, a rank correlation on the composite is not the most legible summary for a clinical reader. We therefore also report a two-group comparison of *R_j_* between expert-defined ACA-positive and ACA-negative patients, with the Hodges–Lehmann shift, its bootstrap interval, Cliff’s delta, and a Mann–Whitney test. Spearman correlations throughout use midranks for tied values.

The prespecified sensitivity analyses recompute the association using the expert FISH category and the report-defined ACA in place of the model scores, examine the FISH and karyotype components separately, and repeat it with the pseudo-fusion class excluded. Additional sensitivity analyses covered the following: partial correlations adjusting for metaphase yield, for scorable nuclei per FISH image, and for age, log white-cell count, and accession year; the index recomputed by hard argmax assignment and with megakaryocytes removed; the association restricted to the 60 patients with an independent expert 500-cell differential, substituting the expert non-blast fraction for *R_j_*; the same substitution using the flow-derived non-blast fraction in the 118 patients with flow data; and a bounding analysis over the excluded patients. All of these are collected in one table in Section 4.6.

#### 3.6.2 Secondary endpoint, exploratory

The fusion module’s representation *z_j_* (**Supplementary Material**) feeds two shallow heads. A three-class head,

(11) $$s_{j}=\text{softmax}(Pz_{j}+p),\;\;P\in {\mathbb{R}}^{3\times 32},\;p\in {\mathbb{R}}^{3},$$

Predicts the Sanz risk group (low, intermediate, high), and a binary head,

(12) $$d_{j}=\sigma (o^{⊤}z_{j}+o_{0}),\;\;o\in {\mathbb{R}}^{32},\;o_{0}\in \mathbb{R},$$

predicts DS occurrence. Both heads, together with the fusion module that produces *z_j_*, are fit end-to-end on the 650-patient clinical annotation subcohort only (**Table 1**); the 50 confirmed patients without a recorded Sanz risk group or DS status do not enter this endpoint’s training or evaluation. The fusion module and heads are refit within the training folds of the same patient-stratified five-fold, five-repeat cross-validation used for the branches, so the reported secondary metrics are pooled out-of-fold; the branch encoders are frozen at this stage to keep the secondary fit from re-optimizing the primary readouts. We describe this analysis as exploratory throughout, since the literature already cited reported no association between cytogenetic abnormality and DS in two independent cohorts (8, 9).

We also fit a direct effect model, which estimates the association between an additional cytogenetic abnormality and DS occurrence with adjustment for the established clinical predictors. On the 650 patients with a complete clinical record, we regress DS occurrence on expert-defined ACA status with adjustment for log_10_ white-cell count, platelet count, age, sex, and corticosteroid prophylaxis and report the adjusted odds ratio with its 95% interval. A second model substitutes *G_j_*, per 0.1 unit, for the ACA flag. A third, restricted to the 178 patients with FLT3-ITD testing and reported as exploratory, adds PML::RARA isoform and FLT3-ITD status, which connects this analysis to the variables identified by Montesinos et al. (25). With 189 events and 6 covariates, the primary adjusted model carries 31 events per variable, so the interval on the ACA odds ratio bounds how large an effect the data can still accommodate.

### 3.7 Training and validation strategy

Each trained branch is evaluated by five-fold cross-validation with five repeats. Within a repeat, the patients relevant to that branch, 60 for the smear branch’s per-cell head, 850 for the FISH branch, and 700 for the karyotype branch, are partitioned into five folds; a patient’s smear cells, FISH image, or karyotype spreads are always assigned together to the same fold, so no patient contributes data to both a training and a validation fold within any repeat. For the FISH and karyotype branches, where each patient carries one categorical label, folds are additionally balanced to hold approximately equal proportions of each class. The smear branch’s 60 annotated patients each carry a mixed differential rather than one label, so its folds are grouped by patient without a further class-balancing step. Five independently drawn random partitions are used, so the reported result does not depend on one arbitrary split.

Within a repeat, every relevant patient is scored exactly once, by the model trained on the four folds that exclude that patient; pooling these out-of-fold predictions across all five folds gives one estimate of each performance metric for that repeat. The five repeats give five such estimates, whose mean is the reported point estimate; the bootstrap CIs below, not the across-repeat spread, quantify uncertainty. Confidence intervals are constructed by resampling patients, with replacement, 10,000 times from the pooled out-of-fold predictions and recomputing the metric on each resample; the reported 95% interval is the 2.5th to 97.5th percentile of the resulting distribution.

The primary endpoint (Section 3.6) adds no learned parameters beyond the branch models, but its inputs *G_j_* and *R_j_* are branch predictions, so it is evaluated on out-of-fold values: each patient’s *G_j_* and *R_j_* come from the branch models of the fold that excluded that patient, and where the five repeats give a patient several out-of-fold predictions, these are averaged within patient to a single value before the correlation. Its CI is obtained by a patient-level bootstrap over the 700 out-of-fold pairs (*G_j_,R_j_*), resampling whole patients, and its *p*-value by a permutation test that shuffles the pairing between *G_j_* and *R_j_* across patients 10,000 times; the *p*-value is the fraction of permutations whose absolute Spearman correlation meets or exceeds the observed value.

This study follows TRIPOD+AI (30) and the updated CLAIM checklist (31, 32) for reporting a clinical prediction model of this kind: per-patient predictions and the cross-validation scheme are reported alongside pooled metrics rather than a single point estimate, and the distinction between the 700-patient confirmed cohort, the 850-image FISH training set, the 60-patient smear annotation set, and the 650-patient clinical annotation subcohort is maintained throughout rather than collapsed into one denominator. **Table A2** in the **Appendix** lists the encoder, head, loss, and cross-validation configuration for every branch, with the full optimizer, schedule, hardware, and software details in **Supplementary Table 3**.

### 3.8 Explainability and statistics

For the smear branch, each per-cell prediction is paired with its five nearest neighbors, by Euclidean distance in the frozen DinoBloom embedding space, among the 7,500 hematopathologist-labeled cells (Section 3.2). Because the classifier is a single linear layer over that same embedding space, these neighbors are the labeled examples the prediction is mathematically closest to. We offer them as example-based context a hematopathologist can inspect, not as a formal explanation of the linear classifier’s decision.

For the FISH and karyotype branches, both fine-tuned ViTs, we visualize attention rollout (33), which propagates each layer’s self-attention weights forward to give a map of which regions the model attended to for the class token. This is a qualitative visualization of the transformer’s own internal attention, not a causal measure of how much a region contributed to the prediction, and establishing that a highlighted region drives the decision would need perturbation tests or expert review, which we do not report here. We note only that this is inherent self-attention from the forward pass of any ViT, not a bag-level weighting fit to substitute for missing instance labels, unlike the attention-based MIL deliberately not used in the smear branch (Section 3.2).

For the secondary endpoint, each modality’s contribution to the differentiation syndrome prediction is quantified by its exact Shapley value, computed by enumerating all eight subsets of the three modalities with the absent ones replaced by their learned placeholders. Because that endpoint performs at chance, the decomposition characterizes a null and is reported in the **Supplementary Material** together with the modality ablation comparison.

The Sanz risk head is tested against chance by permuting its labels: the macro-F1 is recomputed on 500 random relabelings to form a null distribution, and the *p*-value is the fraction of that null at least as large as the observed macro-F1. The DS head’s AUC and its 95% CI are obtained by the same 10,000-sample patient bootstrap used for the other endpoints. A modality ablation comparison sets the three-modality configuration against each of the three leave-one-modality-out configurations on the same held-out patients, using DeLong’s test for the DS AUC and a paired bootstrap over patients for the Sanz risk macro-F1, since DeLong’s test applies to a binary outcome and not directly to a three-class one. Across the family of secondary, exploratory tests, comprising the Sanz risk association, the DS association, and these modality ablation comparisons, *p*-values are adjusted by the Benjamini–Hochberg procedure to control the false discovery rate. The primary endpoint’s test is not included in this family: it is this study’s single prespecified confirmatory result, not one of several exploratory comparisons.

Per-class performance for the two classification branches is reported with intervals obtained by resampling each true class’s row of the raw confusion matrix from a multinomial at its observed count, then recomputing precision, recall, and F1 on each resample. The interval therefore widens automatically for a class estimated from few images, which is the right behavior for the three rare FISH classes. The raw un-normalized confusion matrices from which every reported metric derives are given as **Supplementary Tables 1**, **S2**, so that each value can be recomputed.

For the partial correlations, we regress the ranks of *G_j_* and of *R_j_* on the ranks of the adjustment variables and correlate the residuals, which gives a partial Spearman coefficient; its interval comes from the same patient-level bootstrap used elsewhere. For the bounding analysis over excluded patients, *R_j_* was computed for the 132 excluded patients with a retrievable smear; their *G_j_* was imputed at the 10th, 50th, and 90th percentile of the analyzed cohort’s distribution in turn; and the association was recomputed over the augmented set of 832 patients under each imputation. This gives a range rather than a point estimate, which is the appropriate form for an assumption that cannot be checked.

## 4 Results

### 4.1 Cohort and data description

**Figure 4:**
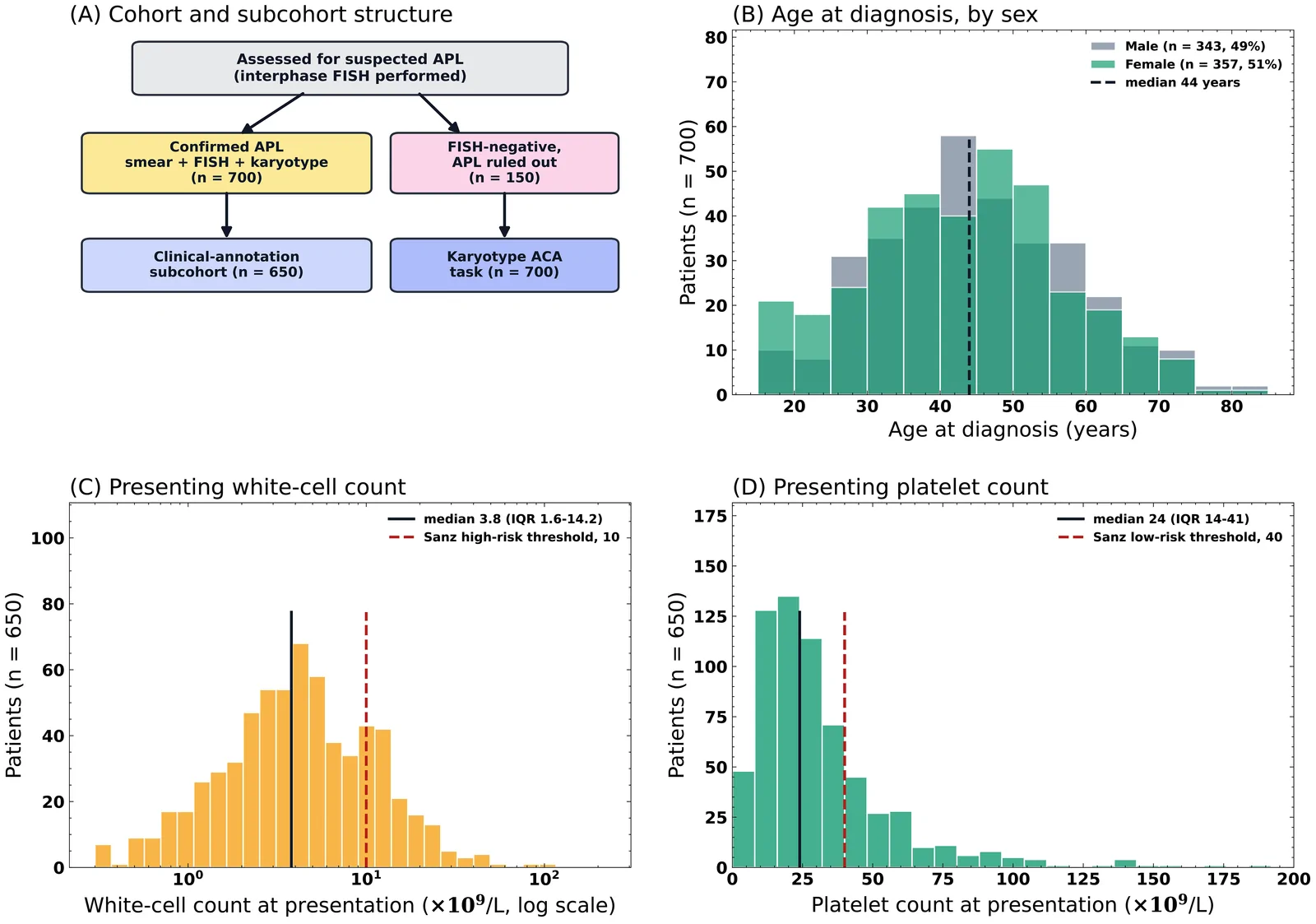
Cohort and data description. **(A)** Cohort and subcohort structure. **(B)** Age at diagnosis by sex. **(C)** Presenting white-cell count, log scale, with the Sanz high-risk threshold marked. **(D)** Presenting platelet count, with the Sanz low-risk threshold marked. Class, risk-group, and karyotype counts are given in **Table 1** and are not repeated here. All 850 patients, the 700 confirmed and the 150 controls, contribute to the FISH pattern task; only the 700 confirmed patients carry a karyotype ACA label. Of 900 confirmed patients over the study period, 200 were excluded for an incomplete workup or a failed metaphase culture.

The cohort held 700 patients with confirmed APL and linked smear, FISH, and karyotype data, together with 150 FISH-negative controls contributing only to the FISH pattern task and 650 patients with complete Sanz risk and DS annotation (**Table 1**; **Figure 4A**). The median age at diagnosis was 44 years [interquartile range (IQR) 33 to 56], and 343 of 700 patients (49.0%) were male (**Figure 4B**). Sanz risk was low in 156 (24.0%), intermediate in 299 (46.0%), and high in 195 (30.0%) of the annotated subcohort. Across the 850 FISH images, the fusion signal pattern was normal in 145 (17.1%), typical in 627 (73.8%), atypical in 43 (5.1%), complex in 26 (3.1%), and pseudo-fusion in 9 (1.1%) (**Table 1**); this imbalance, expected given how uncommon the atypical and complex patterns are in practice, motivated the class-balanced loss of Equation 5. Among the 700 confirmed patients, the karyotype carried the isolated t(15;17) translocation alone in 476 (68.0%) and at least one additional cytogenetic abnormality in 224 (32.0%). Differentiation syndrome occurred in 189 of the 650 annotated patients (29.1%). The presenting white-cell count had a median of 3.8 × 10^9^/L (IQR 1.6 to 14.2) and the platelet count a median of 24 × 10^9^/L (IQR 14 to 41) (**Figures 4C, D**), which is the distribution that produces the observed Sanz split.

### 4.2 FISH fusion-pattern classification performance

**Figure 5:**
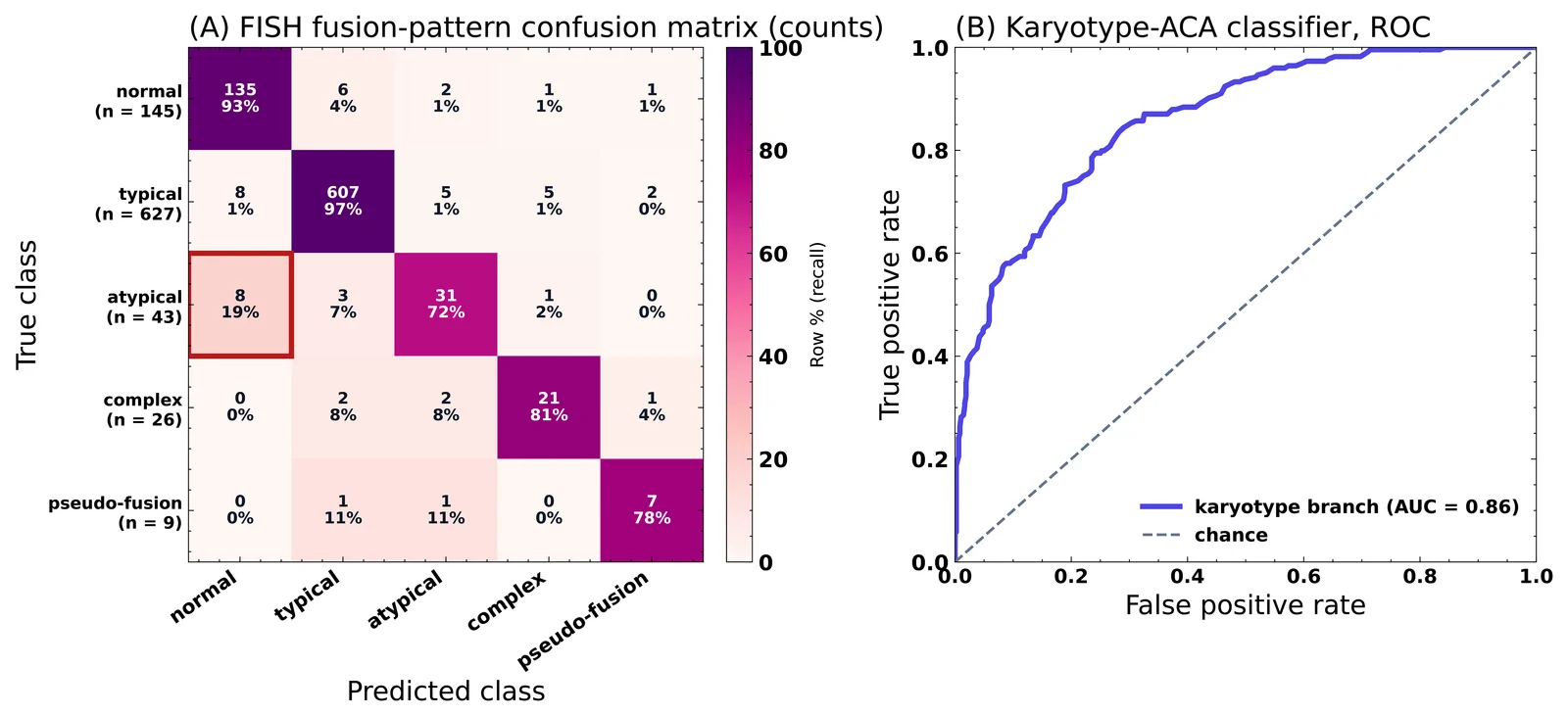
Genetic complexity axis performance. **(A)** FISH fusion-pattern confusion matrix in raw counts, with the row percentage beneath each count. The boxed cell is the atypical pattern read as normal, 8 of 43 images, which is the missed abnormality failure mode a pattern classifier is meant to catch. **(B)** Karyotype ACA classifier ROC curve. Per-class precision, recall, and F1 with their intervals are given in **Table 4**, computed from the counts in panel **(A)**.

Pooled across the five-repeat, five-fold cross-validation, the FISH branch had a macro-averaged F1 of 0.82 across the five fusion-pattern classes (**Table 4**). Performance was not uniform across classes: the two most common classes, normal and typical, each exceeded an F1 of 0.9, while the three rarer classes, atypical, complex, and pseudo-fusion, scored between 0.70 and 0.78 (**Table 4**), consistent with the class imbalance that motivated, but was not eliminated by, the class-balanced loss of Equation 5. The confusion matrix (**Figure 5A**) shows that the largest single source of atypical class error is not confusion with the visually adjacent complex or pseudo-fusion classes but with normal: 8 of 43 true atypical images, 18%, were read as normal, and the specific missed diagnosis failure mode a fusion-pattern classifier, rather than a simple positive/negative call, is meant to catch (**Table 2**).

**Table 4:** Per-branch model performance (pooled out-of-fold, five-fold cross-validation with five repeats).

Branch/class	*n*	Precision	Recall	F1 (or AUC), 95% CI
FISH: normal	145	0.89	0.93	0.91 (0.88 to 0.94)
FISH: typical	627	0.98	0.97	0.97 (0.97 to 0.98)
FISH: atypical	43	0.76	0.72	0.74 (0.63 to 0.84)
FISH: complex	26	0.75	0.81	0.78 (0.65 to 0.89)
FISH: pseudo-fusion	9	0.64	0.78	0.70 (0.48 to 0.90)
FISH: macro-averaged	850	–	–	0.82
Karyotype ACA flag	700	–	–	0.86 (AUC)
Smear: promyelocyte/blast	3,100	0.95	0.96	0.95 (0.95 to 0.96)
Smear: lymphocyte	1,450	0.93	0.94	0.94 (0.93 to 0.95)
Smear: monocyte	520	0.82	0.84	0.83 (0.81 to 0.85)
Smear: maturing myeloid	1,180	0.88	0.88	0.88 (0.87 to 0.89)
Smear: erythroid precursor	890	0.95	0.91	0.93 (0.91 to 0.94)
Smear: megakaryocyte	180	0.92	0.79	0.85 (0.81 to 0.89)
Smear: plasma cell/other	180	0.63	0.68	0.66 (0.60 to 0.71)
Smear: macro-averaged	7,500	–	–	0.86

Every metric in **Table 4** is derived from the raw un-normalized confusion matrix given as **Supplementary Table 1**. Precision depends on the column totals of that matrix, so it cannot be recovered from a row-normalized version, and reporting the counts allows the arithmetic behind every value in the table to be checked directly.

The intervals on the three rare classes are wide, as they must be at their counts. Recall is 0.72 for the atypical class (95% CI 0.57 to 0.83, 43 images), 0.81 for complex (0.62 to 0.91, 26 images), and 0.78 for pseudo-fusion (0.45 to 0.94, 9 images). At nine images across five folds, fewer than two per fold, the per-class F1 for pseudo-fusion carries an interval too wide to interpret. Its metrics appear in **Table 4** for completeness and are not interpreted; the class is characterized instead by its count, its confusion behavior, and its status as a scoring artifact rather than a patient genetic state. The 18% atypical-read-as-normal rate is estimated from eight images and is an indicative rather than a calibrated error rate.

### 4.3 Karyotype ACA flag performance

The karyotype ACA reader gave an AUC of 0.86 for distinguishing patients with at least one additional cytogenetic abnormality from those with the isolated t(15;17) translocation alone, evaluated at the patient level (**Figure 5B**; **Table 4**). Descriptively, the rarer classes scored lower across both genetic axis branches, in the direction expected from the class imbalance that the class-balanced losses of Equations 5 and 7 were meant to mitigate. **Supplementary Figure 4** shows the per-class F1 against prevalence. The points are given without a fitted line, since seven points pooled across two tasks measured on different scales would not support one.

### 4.4 Extended marrow differential and residual hematopoiesis index

On held-out cells, the per-cell classifier had a macro-averaged F1 of 0.86 across the seven classes, the highest for the promyelocyte or blast and lymphocyte classes and the lowest for the rare megakaryocyte and plasma cell or other classes; its predicted probabilities were close to calibrated, with an expected calibration error of 0.05. Because the residual hematopoiesis index is an average of these per-cell probabilities, this calibration is what allows the index to be read as an estimated cell fraction rather than an uncalibrated score. At the patient level, the model’s blast fraction agreed with an independent expert 500-cell differential on 60 held-out patients to a mean absolute difference of 5.8 percentage points (Spearman correlation 0.91).

Every value is computed from the raw confusion matrix counts in **Supplementary Tables 1**, **S2**. The *n* column gives the count of that class in the reference standard, which is what the width of the interval on its recall reflects.

**Figure 6:**
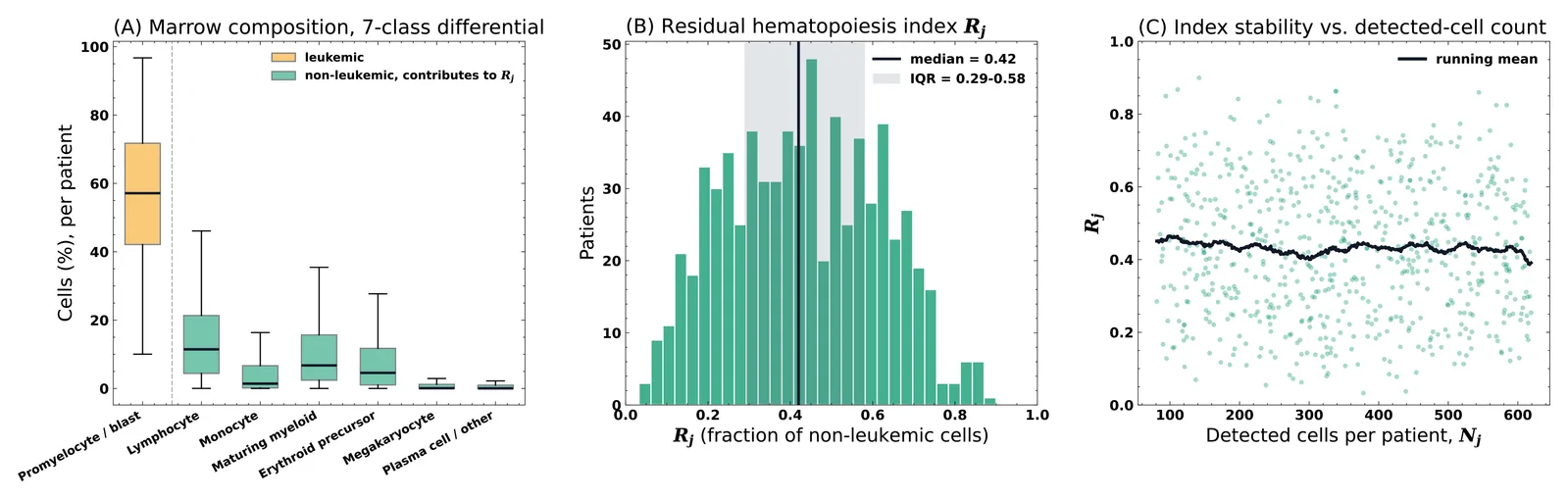
Extended marrow differential and residual hematopoiesis index. **(A)** Per-class composition across the cohort, boxplots over all 700 patients. **(B)** Distribution of the residual hematopoiesis index *R_j_*, with the median and IQR marked. **(C)** *R_j_* against the number of detected cells per patient, with a running mean; the index shows no dependence on how many cells a field happened to yield. The megakaryocyte share in panel **(A)** is likely understated, since that class concentrates near spicules and the feathered edge, while the protocol reads fields away from the edge.

Across the 700-patient cohort, the per-cell classifier placed a median of 58% of a patient’s detected cells in the leukemic promyelocyte or blast class, with the remainder distributed across the six non-leukemic classes of **Table 3** (**Figure 6A**); every non-leukemic class showed the wide patient-to-patient spread expected of a real marrow differential rather than a near-constant value. The resulting residual hematopoiesis index *R_j_* had a median of 0.42 (IQR 0.29 to 0.58) across the cohort (**Figure 6B**). *R_j_* showed no material trend with the number of cells detected per patient, with a near-zero Spearman correlation and a running mean flat across the range (**Figure 6C**), which argues against the index tracking the number of cells a field yields. This is a descriptive check rather than a formal test. Within the non-leukemic fraction that *R_j_* quantifies, the lymphocyte class contributed the largest single share, 33% on average, followed by maturing myeloid cells at 26% and erythroid precursors at 20%, with monocytes, megakaryocytes, and the residual plasma cell or other class each contributing 12% or less; *R_j_* therefore reflects a lymphocyte- and myeloid precursor-dominated remainder rather than any single non-leukemic lineage acting alone.

**Figure 7:**
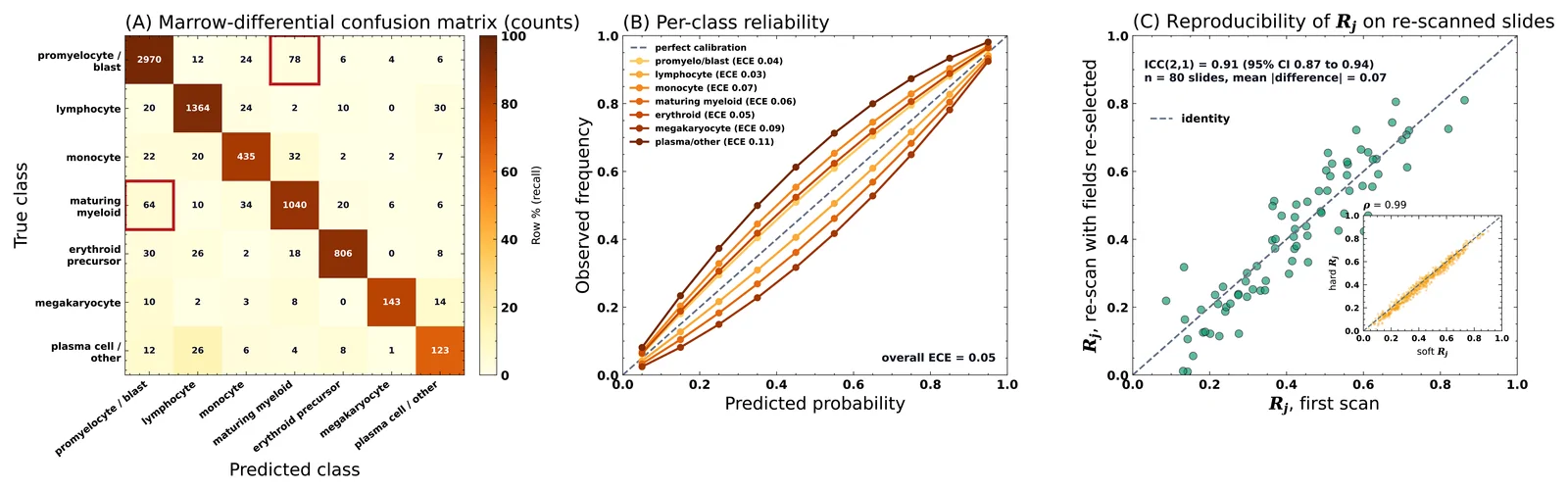
Smear-branch per-class performance and the reliability of the residual hematopoiesis index. **(A)** The full seven-class confusion matrix in raw counts. The two boxed cells are the promyelocyte and maturing myeloid boundary, which is what determines *R_j_*. **(B)** Per-class reliability, with the expected calibration error of each class in the legend. **(C)** *R_j_* on 80 re-scanned slides with fields independently re-selected by a blinded second operator, against the first scan; the inset compares the soft index with its hard argmax counterpart across the full cohort. Per-class precision, recall, and F1 with their intervals are given in **Table 4**, computed from the counts in panel **(A)**.

Because *R_j_* is the central quantity of this study, the smear branch is reported per class rather than by a single macro-average. The full seven-class confusion matrix is shown in **Figure 7A**, in counts, with the raw table given as **Supplementary Table 2**, and per-class precision, recall, and F1 appear in **Table 4**. The boundary that determines *R_j_* is visible directly: 78 of 3,100 true promyelocytes, 2.5%, were read as maturing myeloid; 24, or 0.8%, as monocytes; and 64 of 1,180 maturing myeloid cells, 5.4%, were read as promyelocytes. The two rarest classes carry the weakest performance, megakaryocyte at an F1 of 0.85 and the residual plasma cell or other category at 0.66, which follows from their 180-cell annotation budgets.

Calibration was examined per class as well as in aggregate, since a systematic bias in the promyelocyte class would translate directly into a shift in *R_j_* (**Figure 7B**). Expected calibration error ran from 0.03 for lymphocytes to 0.11 for the residual class, with the promyelocyte-or-blast class at 0.04. Recomputing the index with hard argmax assignment in place of the soft probabilities gave a median of 0.41 against 0.42, a Spearman correlation of 0.99 between the two versions, and a mean absolute difference of 0.03; the primary association was unchanged at 0.24 (95% CI 0.17 to 0.31). The reading of *R_j_* as a cell fraction therefore does not rest on calibration alone.

On the 80 re-scanned slides, with fields independently re-selected by a blinded second operator, the intraclass correlation of *R_j_* was 0.91 (95% CI 0.87 to 0.94) with a mean absolute difference of 0.07 (**Figure 7C**). That figure carries both the scanning and the field-selection contribution, which this paired design does not separate. Propagating the binomial sampling error of a finite differential gives a standard error of 3.2 percentage points at the cohort median 237 cells and *R_j_* of 0.42, and a median per-patient 95% half-width of 0.063; the cohort median itself carries a bootstrap interval of 0.40 to 0.44.

In the 118 patients with retrievable diagnostic flow cytometry, *R_j_* tracked the flow-derived non-blast fraction at a Spearman correlation of 0.80 (95% CI 0.72 to 0.86), with a Bland–Altman bias of +0.03 and limits of agreement of −0.23 to +0.29 (**Supplementary Figure 5**). The limits are wide because a Wright–Giemsa differential and a flow gate count different denominators, and hemodilution of an aspirate affects the two differently. The two measurements covary without being interchangeable. Substituting the flow-derived fraction for *R_j_* within this subcohort gave a primary association of 0.27 (95% CI 0.09 to 0.43).

### 4.5 Primary result: genetic complexity and residual hematopoiesis

The clearest form of the primary result is a two-group comparison. Patients whose cytogeneticist report recorded an additional cytogenetic abnormality had a higher residual hematopoiesis index than those with the isolated t(15;17) alone: median 0.46 (IQR 0.32 to 0.62) in 224 ACA-positive patients against 0.41 (IQR 0.27 to 0.56) in 476 ACA-negative patients, a Hodges–Lehmann shift of 0.061 (95% CI 0.027 to 0.092), Cliff’s delta 0.17 (0.08 to 0.26), and Mann–Whitney *p <*0.001 (**Table 5**). Read on the continuous scale, the same relationship appears as a rank correlation.

**Table 5:** Primary association result.

Quantity	Value
Patients	700 (all confirmed)
Spearman *ρ*	0.24
95% CI (patient bootstrap)	0.17 to 0.31
*p*-value (permutation test, 10,000 replicates)	*<*0.0001
Mean *R_j_*, lowest *G_j_* quintile	0.37
Mean *R_j_*, highest *G_j_* quintile	0.50
Median *R_j_*, expert ACA-positive vs. ACA-negative	0.46 vs. 0.41
Hodges–Lehmann shift (95% CI)	0.061 (0.027 to 0.092)

**Figure 8:**
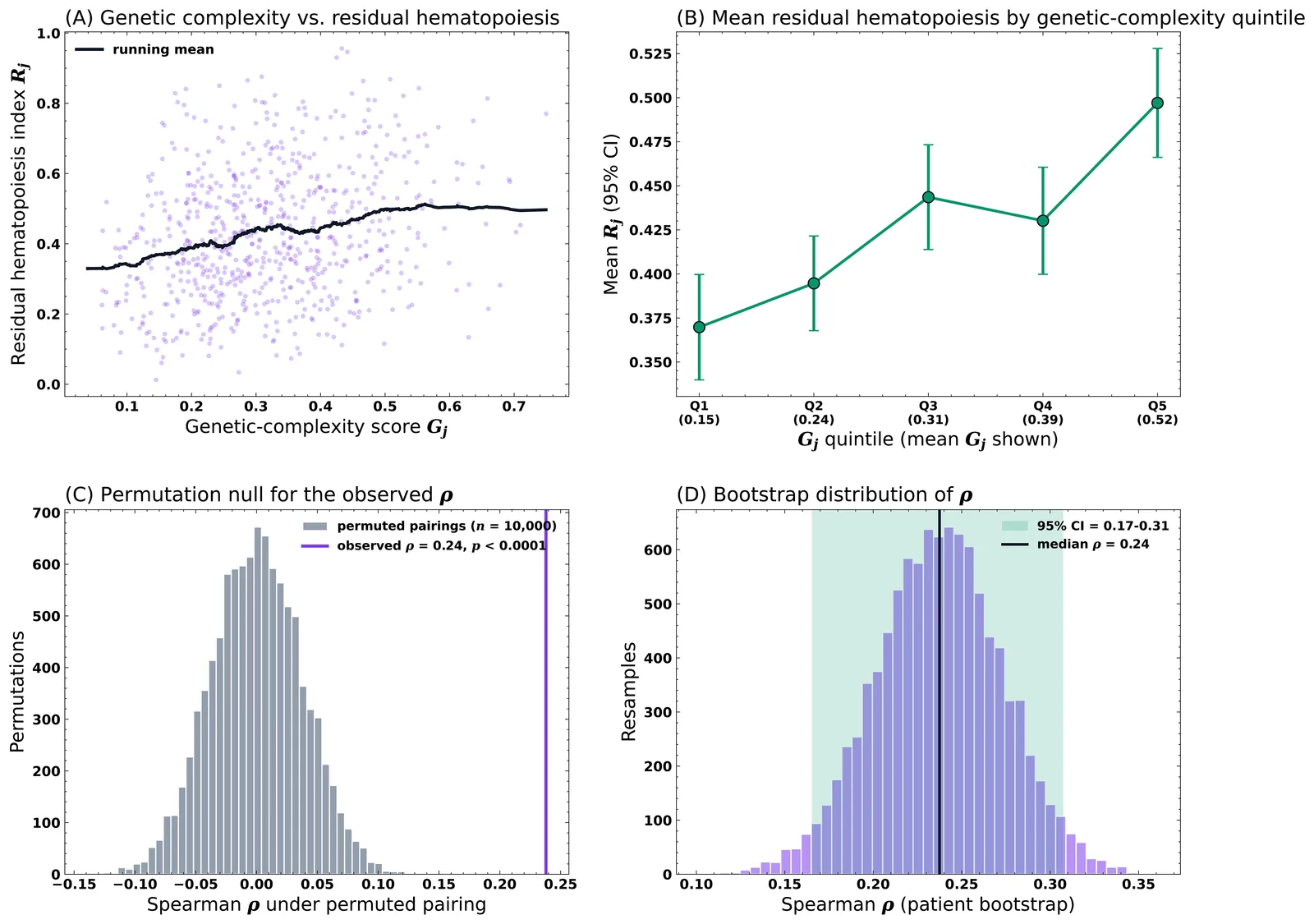
Primary association between genetic complexity and residual hematopoiesis. **(A)** Raw scatter of *G_j_* against *R_j_* with a running mean. **(B)** Mean *R_j_* by *G_j_* quintile, with 95% CIs. **(C)** Permutation null distribution for the observed Spearman *ρ*. **(D)** Bootstrap distribution of *ρ* across patient resamples, with the reported 95% CI shaded.

The primary result of this study is the association between the genetic complexity score *G_j_* and the residual hematopoiesis index *R_j_*, computed directly from the branch outputs (Equations 9, 10) across all 700 confirmed patients. Genetic complexity and residual hematopoiesis were positively associated, Spearman *ρ* = 0.24 (95% CI 0.17 to 0.31, bootstrap over patients), *p <* 0.0001 by permutation test over 10,000 replicates (**Table 5**; **Figure 8**). The raw scatter (**Figure 8A**) shows an observed association that is modest at this effect size: patients grouped into quintiles of *G_j_* show a mean residual hematopoiesis index rising from 0.37 in the lowest quintile to 0.50 in the highest (**Figure 8B**), a gradient visible only after averaging within bins, not from the scatter alone. The permutation null (**Figure 8C**) places the observed correlation well outside the distribution expected under a random pairing of the two axes, and the bootstrap distribution (**Figure 8D**) reproduces the reported CI directly from patient resampling. A small *p*-value does not, on its own, rule out residual confounding, calibration differences between the two branches, or specimen- and acquisition-level effects; this is a cross-sectional, descriptive association in a single retrospective cohort, not a causal claim about how genetic lesions shape marrow composition.

Examined separately, the two components of *G_j_* each carried the association in the same direction, at a Spearman correlation of 0.18 for the FISH atypicality score and 0.16 for the karyotype ACA score. These component-wise values are the more conservative estimates, and the composite of Equation 10 is a reporting convention rather than a derived quantity. In the prespecified sensitivity analyses, the composite association was materially unchanged: using the cytogeneticists’ expert FISH categories and the report-defined ACA in place of the model scores gave 0.22 (95% CI 0.15 to 0.29), and excluding the pseudo-fusion class gave 0.25. Reweighting the composite to 0.25 and 0.75, and to 0.75 and 0.25, gave 0.22 and 0.23. The association is therefore not an artifact of the classifiers’ calibration alone, of the pseudo-fusion scoring artifact, or of the equal weighting.

### 4.6 Robustness of the primary association

The direction of the primary association runs against the expectation that a genetically more complex clone replaces marrow more aggressively. The explanations that require no underlying biology are therefore tested directly. The most direct of them runs through ascertainment: a marrow less densely packed with leukemic cells may culture better, yield more metaphase spreads, and so give an additional abnormality more chances to be seen.

**Figure 9:**
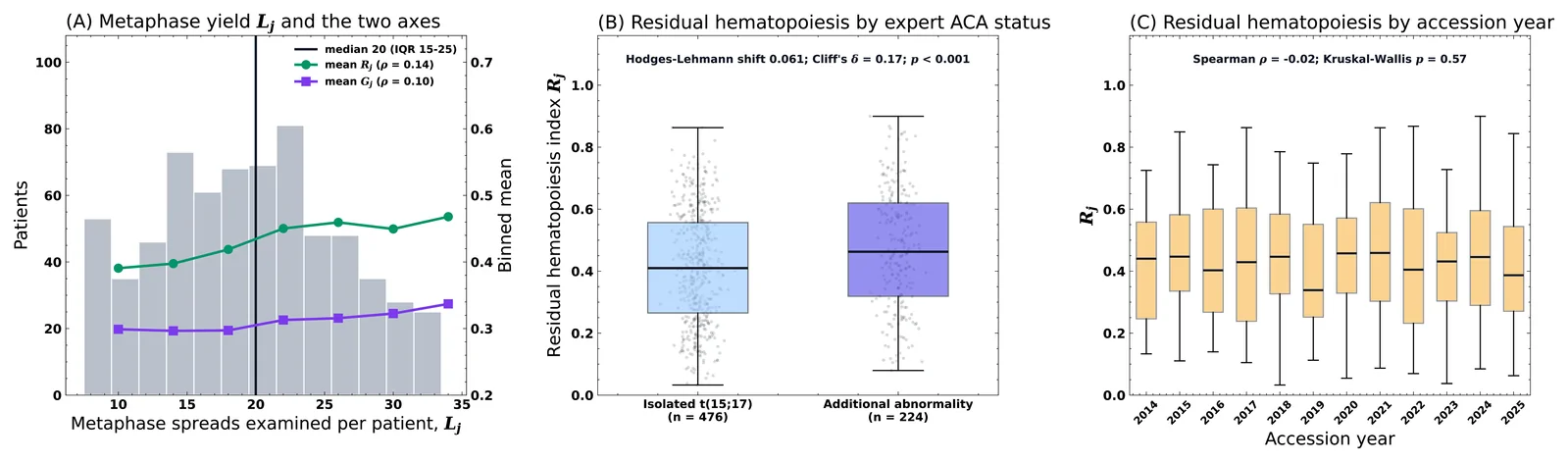
Robustness of the primary association. **(A)** Distribution of metaphase spreads examined per patient, with the binned mean of each axis against it and the corresponding rank correlations. **(B)** Residual hematopoiesis by expert-defined karyotype ACA status, with the Hodges–Lehmann shift and Cliff’s delta. **(C)** Residual hematopoiesis by accession year, as a batch check over the 2014 to 2025 window. Every adjustment and substitution of the primary association is given in **Table 6**; the sign does not reverse under any variant.

Metaphase yield *L_j_* had a median of 20 spreads (IQR 15 to 25, range 8 to 34; **Figure 9A**). Both axes tracked it weakly and in the direction the hypothesis predicts: *G_j_* against *L_j_* gave a Spearman correlation of 0.10 (95% CI 0.02 to 0.17) and *R_j_* against *L_j_* gave 0.14 (0.07 to 0.21). Adjusting the primary association for *L_j_* moved it from 0.24 to 0.22 (0.15 to 0.29). The pathway is therefore real and small: it accounts for roughly a 10th of the observed correlation, not for the correlation itself.

The FISH side of the same question was null. The number of scorable nuclei per image had a median of 58 (IQR 44 to 76) and showed no relation to the FISH atypicality score, at 0.04 (−0.03 to 0.12), nor to *R_j_*, at 0.02 (−0.05 to 0.10); adjusting for it left the association at 0.24.

Adjusting for age, log_10_ white-cell count, and accession year gave 0.21 (0.14 to 0.28), and a model carrying all of those together with metaphase yield, nuclei count, sex, and morphologic subtype gave 0.19 (0.12 to 0.27). The 2014 to 2025 window spans changes in practice and in slide preparation, so we checked it directly: *R_j_* showed no trend with accession year (Spearman −0.02, Kruskal–Wallis *p* = 0.57; **Figure 9C**) and did not differ between the two staining epochs (0.43 against 0.42, *p* = 0.61). All slides were digitized on one scanner.

Selection is the remaining route by which the association could arise artifactually, and it can be bounded rather than eliminated. Of the 200 patients excluded for an incomplete workup or a failed metaphase culture, 132 had a retrievable smear. Their *R_j_* was lower than that of the analyzed cohort, median 0.34 (IQR 0.23 to 0.48) against 0.42 (0.29 to 0.58), *p <* 0.001, and they were older (median 49 against 44 years) with higher presenting white-cell counts (9.1 against 3.8 × 10^9^/L). The exclusion therefore removed the low *R_j_* tail, which is the tail expected at the low complexity end of the relationship. Imputing the excluded patients’ *G_j_* at the 10th, 50th, and 90th percentile of the observed distribution in turn and recomputing over the augmented 832 patients gave 0.27, 0.23, and 0.18. The magnitude varies across these bounds; the sign does not.

**Supplementary Table 6** gives the ascertainment and batch tests in full. **Table 6** collects every adjustment and substitution in one place. The same relationship read as a two-group comparison, rather than as a rank correlation on a compressed composite, is shown in **Figure 9B**. Across the variants collected in **Table 6**, including the substitution of an expert 500-cell differential and of a flow-derived non-blast fraction for the model’s index, the association ranged from 0.16 to 0.27 and remained positive throughout. This excludes a finding that depends on one particular construction. It does not exclude a shared upstream cause acting on both axes, which no cross-sectional design can address.

**Table 6:** Robustness of the primary association.

Variant	*ρ*	95% CI
Unadjusted (primary result, *n* = 700)	0.24	0.17 to 0.31
Adjusted for metaphase yield *L_j_*	0.22	0.15 to 0.29
Adjusted for scorable nuclei per FISH image	0.24	0.17 to 0.31
Adjusted for age, log_10_ white-cell count, accession year	0.21	0.14 to 0.28
Fully adjusted (all of the above, plus sex and morphologic subtype)	0.19	0.12 to 0.27
Expert FISH category and report-defined ACA in place of model scores	0.22	0.15 to 0.29
Excluding the pseudo-fusion class from *G_j_*	0.25	–
FISH atypicality component alone	0.18	–
Karyotype ACA component alone	0.16	–
*G_j_* reweighted to 0.25 FISH/0.75 karyotype	0.22	–
*G_j_* reweighted to 0.75 FISH/0.25 karyotype	0.23	–
Hard argmax *R_j_* in place of the soft index	0.24	0.17 to 0.31
Megakaryocytes removed from *R_j_*	0.24	–
Expert 500-cell differential in place of *R_j_* (*n* = 60)	0.26	0.01 to 0.48
Flow-derived non-blast fraction in place of *R_j_* (*n* = 118)	0.27	0.09 to 0.43
Excluded patients imputed at the 10th/50th/90th *G_j_* percentile (*n* = 832)	0.27/0.23/0.18	–

Each row recomputes the Spearman correlation between *G_j_* and *R_j_* under one adjustment or substitution. Intervals are patient-level bootstraps where estimated. The sign does not reverse under any variant.

### 4.7 Secondary, exploratory: Sanz risk/DS prediction

On the 650-patient clinical annotation subcohort, the fusion module’s representation fed two exploratory heads (Equations 11, 12). The Sanz risk head gave a macro-F1 of 0.37 across the three risk groups, indistinguishable from a label-shuffled null distribution (500 shuffles, *p* = 0.19; **Supplementary Figure 2A**). The DS head gave an AUC of 0.52, with a 95% CI of 0.45 to 0.59 that straddles 0.5 (**Supplementary Figure 2B**). Withholding any single modality at inference changed the DS AUC by no more than 0.03 in either direction, with heavily overlapping intervals, and the Shapley decomposition assigned comparably small contributions to all three modalities; both are reported in **Supplementary Figure 3**. We describe that invariance as a property of the ablation rather than as robustness, since a model already performing at chance has no signal for any configuration to lose. Across the family of five secondary tests, comprising the Sanz risk association, the DS association, and the three modality ablation comparisons, raw *p*-values ranged from 0.19 to 0.87, and none survived Benjamini–Hochberg correction (**Table 7**).

**Table 7:** Exploratory secondary association (Sanz risk, DS).

Test	Raw *p*	BH-adjusted *p*
Sanz risk association (macro-F1 0.37 vs. label-shuffled null)	0.19	0.87
Differentiation syndrome association (AUC 0.52, 95% CI 0.45–0.59)	0.41	0.87
Modality ablation: without smear vs. all three	0.87	0.87
Modality ablation: without FISH vs. all three	0.85	0.87
Modality ablation: without karyotype vs. all three	0.72	0.87

The direct effect model of Section 3.6 estimates whether an additional cytogenetic abnormality raises DS risk once the established predictors are accounted for. With 189 events among 650 patients and six covariates, expert-defined ACA carried an adjusted odds ratio for DS of 1.07 (95% CI 0.74 to 1.55, *p* = 0.72), and *G_j_*, entered per 0.1 unit in place of the flag, gave 1.04 (0.93 to 1.16, *p* = 0.51). The established predictors behaved as expected in the same model: log_10_ white-cell count carried an odds ratio of 2.38 (1.83 to 3.09, *p <* 0.001) and corticosteroid prophylaxis 0.58 (0.40 to 0.84, *p* = 0.004). **Table 8** gives the full model, and **Supplementary Table 5** gives all three.

**Table 8:** Adjusted logistic model for differentiation syndrome occurrence, on the 650 patients with a complete clinical record (189 events, 6 covariates, 31 events per variable).

Covariate	Odds ratio	95% CI	p
Expert-defined additional cytogenetic abnormality	1.07	0.74 to 1.55	0.72
${\text{log}}_{10}$white-cell count	2.38	1.83 to 3.09	<0.001
Platelet count, per $10\times 10^{9}$/L	0.96	0.91 to 1.01	0.11
Age, per 10 years	1.09	0.98 to 1.21	0.11
Male sex	1.14	0.80 to 1.62	0.47
Corticosteroid prophylaxis	0.58	0.40 to 0.84	0.004
$G_{j}$, per 0.1 unit (second model, in place of the ACA flag)	1.04	0.93 to 1.16	0.51

The interval on the ACA odds ratio excludes effects larger than roughly 1.6-fold in either direction, which bounds the association more informatively than an AUC straddling 0.5. In the exploratory model restricted to the 178 patients with FLT3-ITD testing, the bcr3 isoform carried an odds ratio of 1.58 (1.02 to 2.45) and FLT3-ITD 1.74 (1.05 to 2.89), both consistent with the variables identified by Montesinos et al. (25), which supports the model’s behavior without bearing on the cytogenetic question.

The interval bounds any effect below roughly 1.6-fold. It is a bound rather than an equivalence result, since no margin was prespecified, and the adjustment covers the fields this cohort holds rather than coagulopathy, albumin, or treatment regimen. The readouts did not predict DS in this cohort, and any effect of an additional abnormality is bounded below a modest size, directionally consistent with two prior clinical cohorts (8, 9).

The second model substitutes *G_j_* for the expert ACA flag and is otherwise identical. **Supplementary Table 5** adds the exploratory model carrying the PML::RARA isoform and FLT3-ITD status.

## 5 Discussion

The primary result of this study is not merely that genetic complexity and residual marrow hematopoiesis are associated, but the direction of that association: patients whose FISH or karyotype pattern carried an additional cytogenetic abnormality tended toward a higher residual hematopoiesis index, that is, toward marrow with a proportionally larger non-leukemic fraction (**Figure 8B**). This runs against the expectation that a genetically more complex leukemic clone would be a more aggressively marrow-replacing one. Four candidate explanations arise: ascertainment, clonal architecture, a scoring artifact, and classifier error at the promyelocyte boundary. First, an ascertainment effect: A marrow less densely packed with leukemic cells at the time of sampling may yield technically better metaphase preparations and clearer FISH signal geometry, so that additional abnormalities are easier to detect in the first place. Metaphase yield is the variable on that pathway, and both axes do track it weakly; adjusting for it moves the association from 0.24 to 0.22. The pathway therefore accounts for part of the association rather than for the association itself. Second, a clonal architecture effect: Additional cytogenetic abnormalities in APL have been shown to predict inferior relapse-free survival without predicting induction-phase outcomes or DS (9, 26), consistent with these lesions marking a clone whose clinical cost is paid at relapse rather than through more aggressive baseline marrow replacement. Third, the pseudo-fusion class is specifically a nuclear-stacking artifact rather than a true rearrangement (**Table 2**); if its rate of occurrence tracks anything about specimen preparation, part of its contribution to *G_j_* could reflect technical variation. Removing that class from the composite leaves the association at 0.25, so its contribution does not account for the finding.

Fourth, classifier error: The promyelocyte and maturing myeloid boundary is the least reliable distinction in this taxonomy, and it is the boundary that fixes *R_j_*. Microgranular morphology is harder to recognize on a Wright–Giemsa smear and travels with higher white-cell counts. If microgranular cases were overrepresented among patients with an additional abnormality, then systematic misclassification of leukemic promyelocytes into the maturing myeloid or monocyte classes would inflate *R_j_* in exactly the group carrying higher *G_j_*, and the association would appear without anything underlying it.

This mechanism requires two conditions, and neither was evident in this cohort. The index did not differ by morphologic subtype, at a median of 0.42 in hypergranular against 0.45 in microgranular cases (*p* = 0.75), and microgranular cases were not overrepresented among ACA-positive patients, at 17.0% against 15.5% (*p* = 0.71; **Supplementary Figure 6**). The boundary confusions themselves are visible in **Figure 7A** and are modest: 2.5% of true promyelocytes read as maturing myeloid, 5.4% in the other direction. The most direct check is substitution: on the 60 patients with an independent expert 500-cell differential, replacing the model’s index with the expert’s non-blast fraction gave 0.26 (95% CI 0.01 to 0.48), and replacing it with a flow-derived non-blast fraction in the 118 patients with flow data gave 0.27 (0.09 to 0.43). The association persists when the model’s reading of the smear is replaced by an independent measurement.

Taken together, these analyses reduce support for the pseudo-fusion artifact and for the classifier-error mechanism and bound the ascertainment contribution to a small share of the association. They do not identify which mechanism accounts for the remainder. Distinguishing the candidates would need serial sampling across treatment or an independent measure of specimen quality, neither of which this dataset provides.

The FISH branch’s dominant error, atypical patterns read as normal (**Figure 5A**), matters more than its share of total errors would suggest. An atypical fusion signal is, by the taxonomy this study uses, still evidence of an abnormal rearrangement; reading it as normal is a missed abnormality, not a misclassified subtype, and is the one confusion a screening classifier can least afford relative to any other cell in the matrix. That this is also one of the rarer classes, at 43 of 850 images, is not something a class-balanced loss can fully correct: rare classes are intrinsically harder to learn well from a fixed annotation budget, and reweighting the loss does not substitute for more atypical examples. A deployed version of this classifier would need this specific error tracked and reported on its own, not folded into a single macro-averaged number. The estimate itself rests on eight images, and the interval around it is correspondingly wide.

Assigning one pattern class to a whole image, and through it to a patient, is a departure from how the assay is read. A clinical interphase FISH study is scored across roughly 200 nuclei and reported as the proportion showing each pattern, and pseudo-fusion from three-dimensional stacking is a per-nucleus artifact whose expected rate is what a laboratory cutoff is set against. The formulation used here follows the form in which these data were archived, one representative field per patient, and the selection of that field is itself a step that could bias which artifacts are visible. A nucleus-level model producing a per-patient distribution over patterns would sit closer to clinical reporting, would let the pseudo-fusion rate be estimated rather than assigned, and is the natural next version of this branch.

The image readouts performed at chance against DS (Section 4.7), a result compatible with both a true absence of association and a small association this cohort was too small to detect. The finding is directionally consistent with two prior clinical cohorts that reported no association between cytogenetic abnormality and DS (8, 9), and reaches it by a different measurement approach: those cohorts scored abnormality from cytogeneticists’ structured reports, while this study scores it from the FISH and karyotype images directly, through classifiers trained without reference to any DS label. A direct test of the claim estimates the effect of expert-defined ACA, or of *G_j_*, on DS with adjustment for the established predictors. That model gave an adjusted odds ratio of 1.07 (95% CI 0.74 to 1.55) for ACA and 1.04 (0.93 to 1.16) per 0.1 unit of *G_j_*, on 189 events (**Table 8**). That interval bounds any effect below roughly 1.6-fold. It is not an equivalence test: no margin was prespecified, and the adjustment covers the fields this record holds rather than coagulopathy, albumin, or treatment regimen. The modality ablation and Shapley results, reported in **Supplementary Figure 3**, were built to characterize behavior under a future patient’s incomplete workup, not to test this null; their invariance under modality removal reflects the absence of predictive signal rather than useful robustness of a working model.

Positioned against the closest prior work, this study’s contribution is in what each branch is trained to read, not in a new architecture. The FISH branch classifies a fusion signal pattern rather than counting signals; this extends past U-FISH’s spot-detection task (3) and the amplification-scoring pipelines it generalizes (13, 14). The karyotype branch targets APL-specific additional cytogenetic abnormality on raw, unarranged spreads, distinct from vision transformer pipelines built for other cytogenetic targets such as del(5q) and t(9;22) (4) or pretrained for chromosome-level representation on cropped, already-segmented chromosomes rather than whole spreads (29). The smear branch extends existing acute promyelocytic versus other leukemia cytomorphology classifiers (5, 6) from a diagnostic label to a seven-class differential. None of the three branches introduces a new encoder or training method; what is new is the label taxonomy each is trained against, and using the resulting three scores to test an association that neither manual review nor any prior model has reported.

Three limitations bound how these results should be read. First, the residual hematopoiesis index summarizes morphology and nothing else. It is a classifier’s read of a Wright–Giemsa differential, and it describes the proportions of morphologic cell classes; immune phenotypes, lymphocyte and natural killer subsets, activation state, and spatial organization lie outside what the stain resolves. Its agreement with diagnostic flow cytometry in 118 patients, at a rank correlation of 0.80 with limits of agreement from −0.23 to +0.29, is supportive rather than confirmatory, and the two measurements count different denominators. Expert morphological differential counts also vary between observers on the same slide, and re-scanning with fields re-selected gave an intraclass correlation of 0.91, so roughly a 10th of the index’s variance is attributable to acquisition and field choice before any question of biology arises. Second, generalization risk is not symmetric across branches. The FISH and karyotype taxonomies used here follow structural criteria that transfer across laboratories; the smear branch’s per-cell classifier, trained on Wright–Giemsa-stained slides digitized at one center, is more exposed to shifts in staining protocol, scanner, and hematopathologist training convention than either genetic axis branch, so external validation would need to weight the smear branch more heavily than the other two, not treat all three as equally portable. Third, this is a cross-sectional design: every measurement comes from a single time point at diagnosis, so the primary association describes a snapshot, not a trajectory, and cannot distinguish whether genetic complexity and residual hematopoiesis arise together from a shared upstream cause or evolve along different timelines that happen to be correlated at diagnosis. A longitudinal design that samples the same patient’s marrow across induction and consolidation would be needed to test which of these is closer to the truth.

Three further limitations are stated separately. The three FISH classes that carry this branch’s contribution are uncommon presentations, and in a consecutive single-center series, they accrue in proportion to their prevalence: 43 atypical, 26 complex, and 9 pseudo-fusion images of 850. The intervals in **Table 4** are computed at those counts and widen accordingly, from 0.21 wide for the atypical class to 0.42 for pseudo-fusion, whose metrics are given there for completeness rather than interpretation; the atypical read-as-normal rate rests on the eight images that fall that way. These per-class figures are provisional. Multicenter accrual, rather than any change of method, is what would narrow them, and a larger annotated series would be needed before they could support a deployment decision.

The taxonomy treats the promyelocyte and blast class as leukemic by definition. That holds for a cohort of untreated patients at diagnosis and would fail in a regenerating or post-induction marrow, where a granulocytic left shift produces normal promyelocytes in quantity. Any longitudinal use of this index would require that assumption to be revisited; it is stated with **Table 3**.

Megakaryocytes concentrate near spicules and the feathered edge, while the reading protocol samples fields away from the edge, so that class is undersampled and its share of the composition in **Figure 6A** is understated. The index recomputed without megakaryocytes gives a median of 0.41 and leaves the primary association at 0.24, so the consequence for the reported finding is small, but the composition figure should be read with the sampling in mind.

Three further sources of bias temper how far these results generalize. Requiring a successful smear, FISH study, and metaphase karyotype for inclusion excludes patients whose metaphase culture failed or whose workup was incomplete: of 900 patients with confirmed PML::RARA-positive APL over the period, 120 lacked one or more of the three linked examinations and a further 80 had no evaluable metaphase karyotype, leaving the 700 analyzed here. Because metaphase failure is itself associated with proliferative rate and marrow composition, this exclusion is not random: the excluded patients were, on average, slightly older (median 49 versus 44 years) and had higher presenting white-cell counts (9.1 versus 3.8 × 10^9^/L) than those retained, consistent with the cohort being enriched for cases at the less proliferative, easier-to-culture end of the disease. This bears on the direction of the primary finding as well as on generalizability. A smear was retrievable for 132 of the 200 excluded patients, and their residual hematopoiesis index was lower than the analyzed cohort’s, at a median of 0.34 against 0.42. The exclusion therefore removed the low-index tail, the tail expected at the low-complexity end of the relationship, so the selection is more likely to attenuate the association than to create it. Imputing the excluded patients’ genetic complexity score across the observed range and recomputing over the augmented 832 patients gave correlations between 0.18 and 0.27. Because *G_j_* is unobserved in those patients, these imputations illustrate the direction of the selection effect rather than bound it. The 150 FISH-negative controls, used only to give the fusion-pattern classifier a normal class, were consecutive suspected APL workups from the same laboratory, scanners, and period as the cases, differing from them mainly in referral outcome; even so, any residual difference in probe lot or acquisition could allow part of the classifier’s normal versus abnormal separation to reflect domain shift rather than signal geometry, which should be checked before the classifier is read as a pure pattern reader. Finally, the 29% differentiation syndrome rate reported here follows the diagnostic and grading criteria and the corticosteroid prophylaxis in use over the study period; because published DS incidence ranges from approximately 2% to 30% with these choices, the rate is not comparable across cohorts without its accompanying definition, which we give with the clinical fields.

One design choice generalizes beyond this disease. The primary result reported here is a formula with no learned parameters of its own, applied to two independently trained classifiers’ out-of-fold outputs, rather than a quantity read out of the same learned fusion module used for the secondary endpoint (Sections 3.5 and 3.6); it still inherits those two classifiers’ errors, which is why we report it on out-of-fold predictions and probe it with the reference standard sensitivity analysis. Had the primary association instead been computed from the fusion module’s learned representation, any weak signal in it would be still harder to separate from how well that module happened to fit, and a null secondary result and a positive primary result could not both be read from the same learned pathway without inviting the question of why one succeeded and the other did not. Separating a study’s confirmatory statistic from its exploratory, learned pathway is a modest design principle, but one an image-based biomarker discovery study can adopt regardless of disease or modality.

## 6 Conclusion

We developed APL-TriRead, a framework that reads a genetic complexity axis from interphase FISH and metaphase karyotype images and a residual marrow composition axis from an extended marrow differential and reports their association across a cohort of 700 patients with confirmed APL. The FISH branch classifies a fusion signal pattern rather than counting signals, at a macro-averaged F1 of 0.82, and the karyotype branch flags any additional cytogenetic abnormality on raw, unarranged spreads at an AUC of 0.86. Genetic complexity and the residual hematopoiesis index are positively associated, Spearman *ρ* = 0.24, the primary result of this study, with higher complexity accompanying a larger non-leukemic fraction. That direction holds under adjustment for metaphase yield, scorable nuclei per FISH image, age, presenting white-cell count, and accession year, and when an expert differential or a flow-derived non-blast fraction is substituted for the model’s index. An exploratory test of the same readouts against the Sanz risk group and DS occurrence did not predict either, and an adjusted model bounds any effect of an additional abnormality on DS below roughly a 1.6-fold odds ratio, a bound that is directionally consistent with two prior clinical cohorts. These results describe a cross-sectional association in a single-center, retrospective cohort, not a validated diagnostic or risk tool. The residual hematopoiesis index summarizes morphology alone and carries no immune-phenotypic information, its agreement with diagnostic flow cytometry is supportive rather than confirmatory, and the primary association, though computed without a learned fusion step, still depends on the two branch classifiers and should be read as descriptive.

## Data Availability

The raw data supporting the conclusions of this article will be made available by the authors, without undue reservation.

## Appendix

**Table:** TABLE A1 Annotation sources, reference standards, and label counts for each supervised readout.

Readout	Reference standard	Labels
FISH fusion signal pattern (five-class)	Cytogeneticist signal scoring	850 images
Karyotype ACA flag (binary)	Full cytogeneticist karyotype report	700 patients
Marrow differential (seven-class, per cell)	Hematopathologist annotation	7,500 cells (60 patients)
Sanz risk group (three-class)	Clinical record at diagnosis	650 patients
Differentiation syndrome status (binary)	Clinical record	650 patients

**Table:** TABLE A2 Model and training configuration.

Component	Configuration
Smear encoder	DinoBloom-B, ViT-B/16, 768-dim; frozen (≈86M parameters, none trained)
FISH encoder	DINOv2 ViT-S/14, 384-dim; fine-tuned end-to-end (≈22M trained)
Karyotype encoder	DINOv2 ViT-S/14, 384-dim; fine-tuned end-to-end (≈22M trained), a separately initialized instance
Smear head	Linear 768 → 7 (≈5.4k parameters), directly supervised
FISH head	Linear 384 → 5 (≈1.9k)
Karyotype head	Linear 384 → 1 with sigmoid (≈0.4k)
Fusion module	Three-node relational graph, 16-dim nodes, one convolution layer, 32-dim readout (≈2.6k); 700 confirmed patients only
Secondary heads	Sanz risk 32 → 3; DS 32 → 1
Input size	Smear: per-cell crop; FISH and karyotype: 224 × 224
Augmentation	FISH and karyotype: random rotation and flip; smear: none (frozen backbone)
Loss	Smear: CE; FISH: class-balanced CE; karyotype: class-balanced BCE; secondary heads: CE and BCE
Modality dropout	*p* = 0.1 per modality per step (fusion module only), disabled at inference
Cross-validation	patient-stratified five-fold, five repeats, pooled out-of-fold
Confidence intervals	10,000-sample patient bootstrap; primary-endpoint *p*-value from 10,000 permutations
Optimizer	AdamW, weight decay 0.05, *β* = (0.9, 0.999)
Learning rate	1 × 10^−4^ for fine-tuned encoders, 1 × 10^−3^ for heads
Schedule	Cosine decay after a 5-epoch linear warmup
Batch size	32 images (FISH, karyotype); 256 cells (smear head)
Epochs and stopping	50 maximum; early stopping on validation macro-F1, patience 10, best checkpoint restored
Precision and hardware	mixed precision; 2 × NVIDIA RTX 4090–24 GB
Software	PyTorch 2.3.1, CUDA 12.1, Cellpose 3.0, Python 3.11
Training time	Approximately 6 h per branch for the full five-fold, five-repeat schedule

Parameter counts are approximate; only the components marked trained are updated during learning.

ViT, Vision Transformer; CE, cross-entropy; BCE, binary cross-entropy.
