# Supplementary material for: Multimodal AI reading of genetic complexity and residual marrow composition in acute promyelocytic leukemia
Source: Front Immunol. 2026 Aug 24;17:1933105. doi: 10.3389/fimmu.2026.1933105 (PMC13547524; doi:10.3389/fimmu.2026.1933105)
Supplement: Supplementary file 1 [file DataSheet1.pdf]

Supplementary Material

1 SUPPLEMENTARY METHODS

1.1 Fusion module for the secondary endpoint

The fusion module (Figure S1) supplies the single learned representation per patient, z_j, on which the secondary, exploratory endpoint of the main text is built (Figure S2). It operates on the 700 confirmed patients only: the karyotype ACA flag, one of its three inputs, is undefined for a patient without a t(15;17) translocation, so the 150 FISH-negative controls do not enter it. The primary endpoint of the study uses neither z_j nor this module, and is computed directly from the three branches' out-of-fold outputs by a formula with no additional learned parameters.

Each patient contributes three raw feature vectors from the branches: $h_j^{\text{sm}} = \pi_j \in \mathbb{R}^7$ (smear composition), $h_j^{\text{fi}} = q_j \in \mathbb{R}^5$ (FISH class probabilities), and $h_j^{\text{ka}} = \rho_j \in \mathbb{R}$ (karyotype ACA score). Each is linearly projected into a shared 16-dimensional space,

$$g_j^m = A_m h_j^m + a_m, \qquad m \in \{\text{sm}, \text{fi}, \text{ka}\}, \tag{S1}$$

with $A_{\text{sm}} \in \mathbb{R}^{16\times7}$, $A_{\text{fi}} \in \mathbb{R}^{16\times5}$, $A_{\text{ka}} \in \mathbb{R}^{16\times1}$, and $a_m \in \mathbb{R}^{16}$. The projections of Equation S1 are the only place the three branch outputs meet a common dimension.

The three projected nodes are joined by two labeled edge types, following a relational graph convolution (Schlichtkrull et al., 2018): a genetic edge connects the FISH and karyotype nodes, and a cross-axis edge connects the smear node to each of the other two. One convolution layer updates every node from its neighbors under each edge type and from itself,

$$g_j'^{\,m} = \text{ReLU}\Big(W_0 g_j^m + \sum_{r:\, N_r(m)\neq\varnothing} \frac{1}{|N_r(m)|} \sum_{m'\in N_r(m)} W_r\, g_j^{m'}\Big), \tag{S2}$$

where $N_{\text{gen}}(\text{fi}) = \{\text{ka}\}$, $N_{\text{gen}}(\text{ka}) = \{\text{fi}\}$, $N_{\text{gen}}(\text{sm}) = \varnothing$, $N_{\text{cross}}(\text{sm}) = \{\text{fi}, \text{ka}\}$, $N_{\text{cross}}(\text{fi}) = N_{\text{cross}}(\text{ka}) = \{\text{sm}\}$, and $W_0, W_{\text{gen}}, W_{\text{cross}} \in \mathbb{R}^{16\times16}$ are the only weight matrices at this stage, shared across all three nodes and across patients. In Equation S2 the relation sum runs only over relations with a nonempty neighborhood for the node in question, so the genetic relation, empty at the smear node, contributes no term there and no division by zero arises. A single convolution layer is used, not a deeper stack, consistent with the small size of this module. The graph is reported as the fusion mechanism used, not as a demonstrated improvement over a plain concatenation of the branch outputs followed by a linear head, which these data do not distinguish it from. The three updated nodes are concatenated and projected to the final fused representation,

$$z_j = U\big[\, g_j'^{\,\text{sm}} \,\|\, g_j'^{\,\text{fi}} \,\|\, g_j'^{\,\text{ka}} \,\big] + u, \qquad U \in \mathbb{R}^{32\times48},\ u \in \mathbb{R}^{32}, \tag{S3}$$

so $z_j \in \mathbb{R}^{32}$ is the representation that Equation S3 hands to the secondary heads.

During training, each of the three raw inputs is independently replaced with a learned, modality-specific placeholder vector with probability 0.1 at each training step; this modality dropout is disabled at inference, since every patient in this cohort has all three branch outputs observed. Its purpose is prospective: a future

patient may present with an incomplete workup, for example a karyotype still pending, and the module is trained to still produce a usable z_j in that case.

1.2 Shapley decomposition for the differentiation-syndrome head

Writing $v(S)$ for the differentiation-syndrome head's predicted probability when only the modalities in $S \subseteq \{\mathrm{sm}, \mathrm{fi}, \mathrm{ka}\}$ are observed and the rest are replaced by the learned modality-dropout placeholder of Section 1.1, modality m's contribution to patient j's prediction is

$$\phi_m(j) = \sum_{S \subseteq \{\mathrm{sm},\mathrm{fi},\mathrm{ka}\} \setminus \{m\}} \frac{|S|!\,(2 - |S|)!}{3!} \big[v(S \cup \{m\}) - v(S)\big]. \tag{S4}$$

With three modalities all eight subsets can be evaluated directly, so Equation S4 is computed by exact enumeration rather than by sampling. Per patient the three $\phi_m(j)$ sum to $v(\{\mathrm{sm}, \mathrm{fi}, \mathrm{ka}\}) - v(\varnothing)$, and we summarize across patients by the mean of $|\phi_m(j)|$. Exactness refers to the enumeration rather than to causal attribution. Because the head performs at chance, the decomposition characterises a null (Figure S3).

2 SUPPLEMENTARY FIGURES

Figure S1 shows the fusion module and its modality-dropout training. Figure S2 gives the secondary, exploratory endpoint it feeds, and Figure S3 the ablation and Shapley decomposition of the same head. Figure S4 plots per-class F1 against class prevalence for the two genetic-axis branches. Figure S5 reports the agreement between the residual hematopoiesis index and the flow-derived non-blast fraction, and Figure S6 the two tests of the classifier-error mechanism.

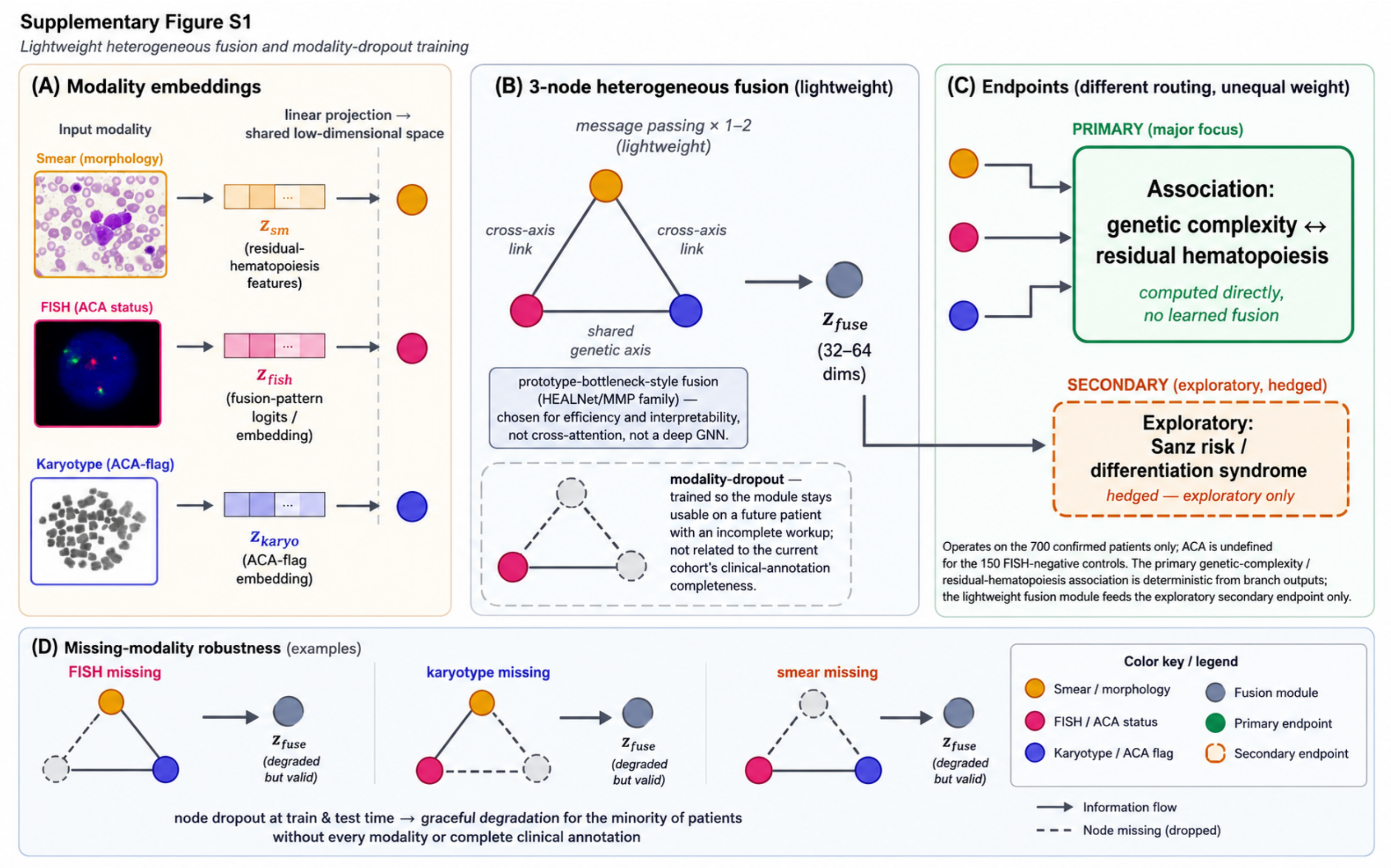

Figure S1. Fusion module and modality-dropout training. (A) Each branch output is linearly projected into a shared low-dimensional space. (B) A three-node relational graph, over the 700 confirmed patients only, passes messages along a shared-genetic edge and two cross-axis edges to a small fused representation. (C) The fused representation feeds only the secondary, exploratory endpoint; the primary genetic-complexity and residual-hematopoiesis association is computed directly from the branch outputs and does not pass through the graph. (D) Modality dropout allows the module to accept a missing modality at inference. The endpoint performs at chance, so this panel describes the training procedure rather than a robustness result.

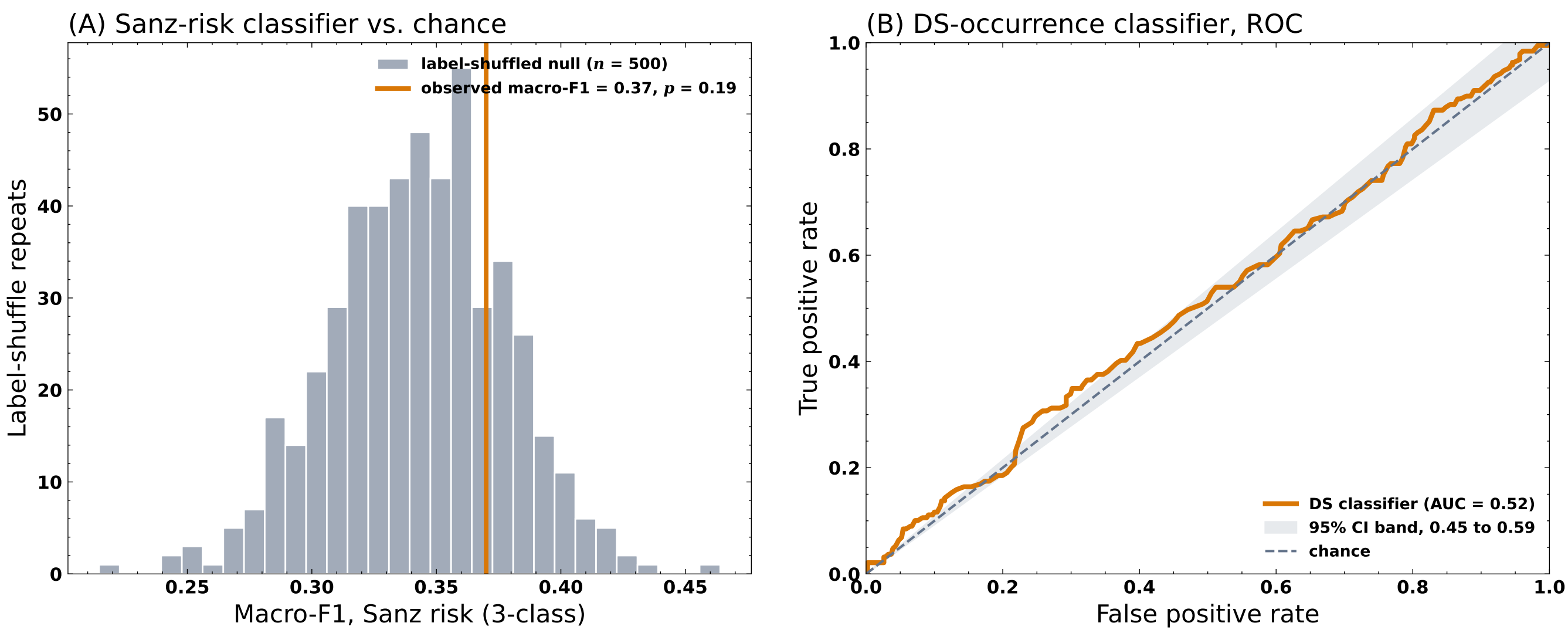

Figure S2. Secondary, exploratory endpoint: Sanz risk and differentiation syndrome. (A) Sanz-risk classifier macro-F1 against a label-shuffled null distribution, from which it is not distinguishable. (B) Differentiation-syndrome classifier ROC, with its 95% confidence band shown against chance. The numerical values are given in Table 7 of the main text, and the adjusted effect model in Table 8 of the main text.

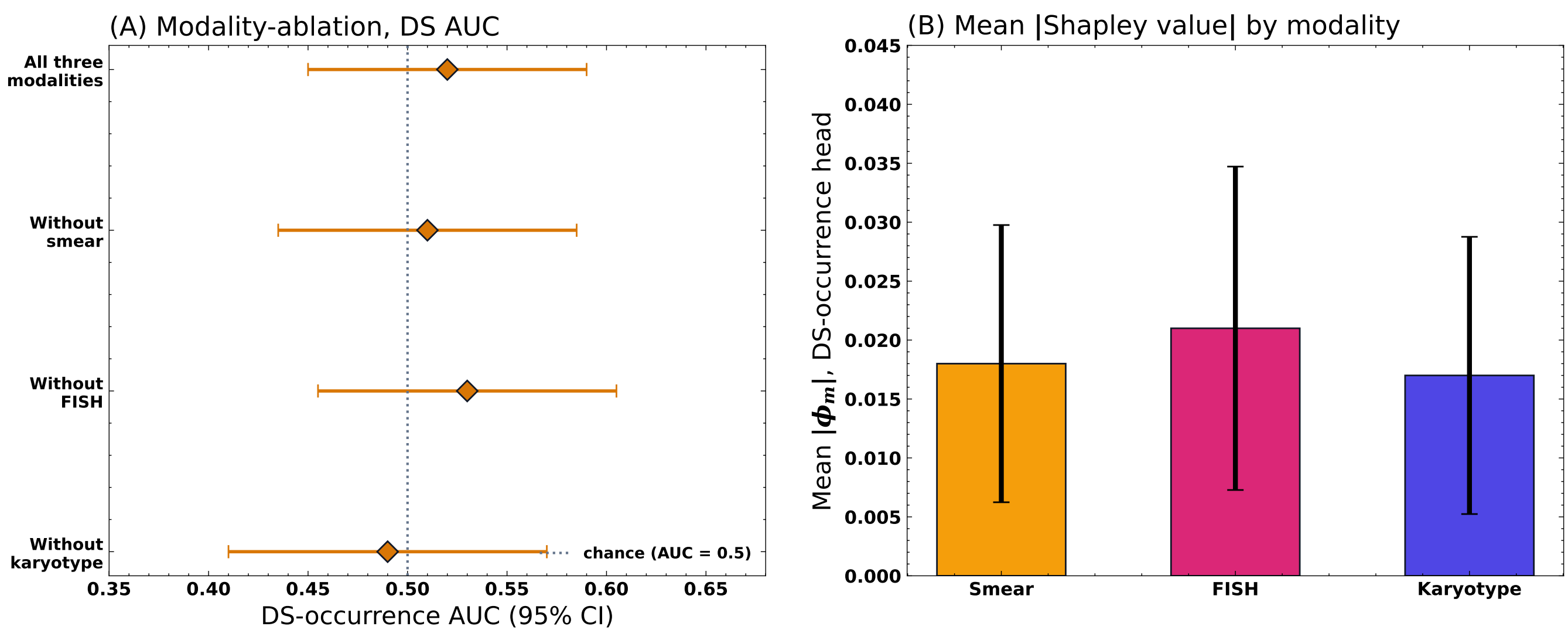

Figure S3. Modality ablation and Shapley decomposition for the differentiation-syndrome head. (A) DS-occurrence AUC under modality ablation, with heavily overlapping intervals across configurations. (B) Mean absolute Shapley value per modality; no modality dominates. Both panels characterise a head that performs at chance, so the invariance under modality removal reflects an absence of predictive signal rather than robustness.

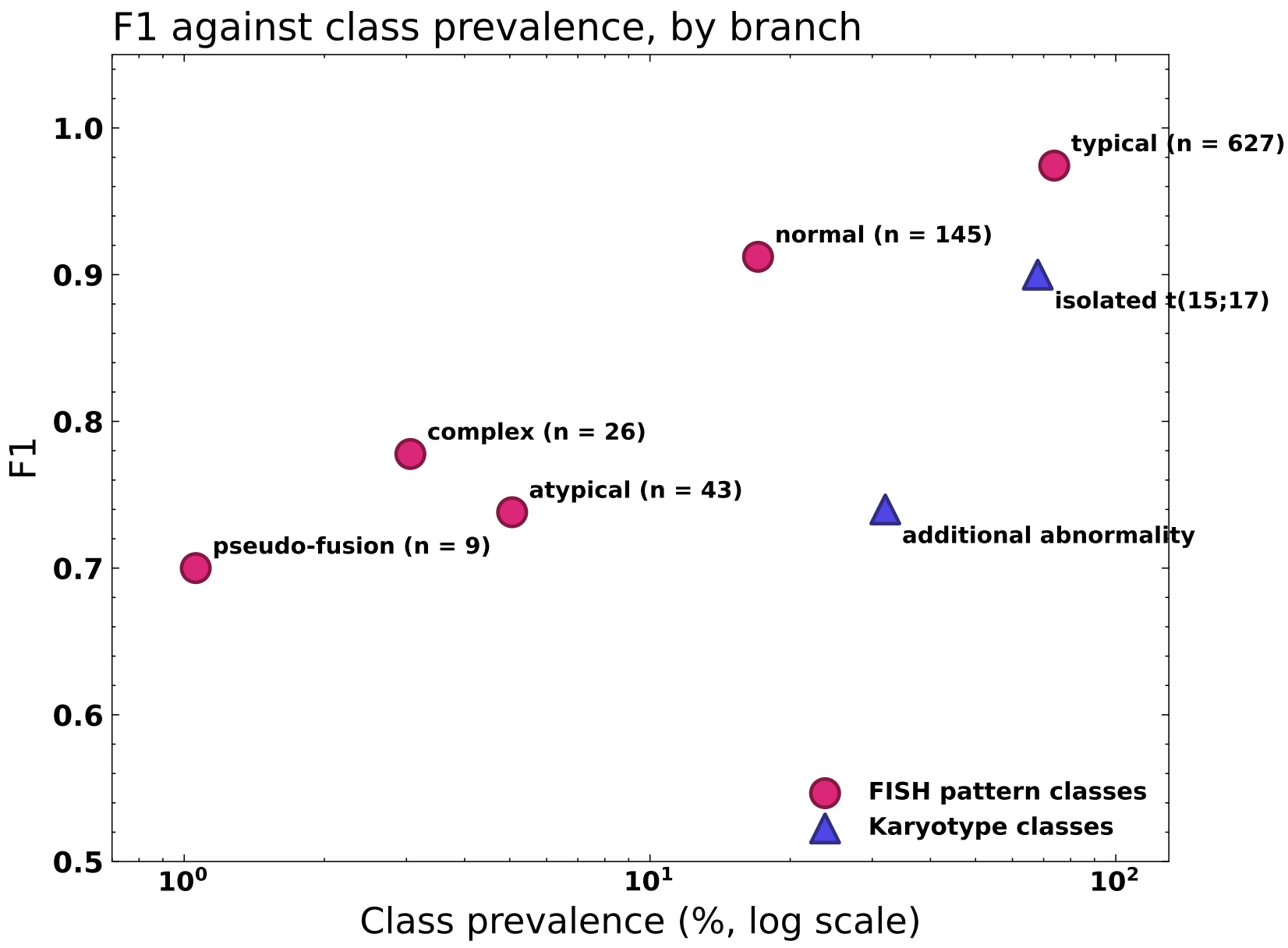

Figure S4. F1 against class prevalence for both genetic-axis branches, on a log scale, with each FISH class labelled by its count. No trend is fitted: the seven points pool two tasks measured on different scales, and a fitted line would not be supported by them.

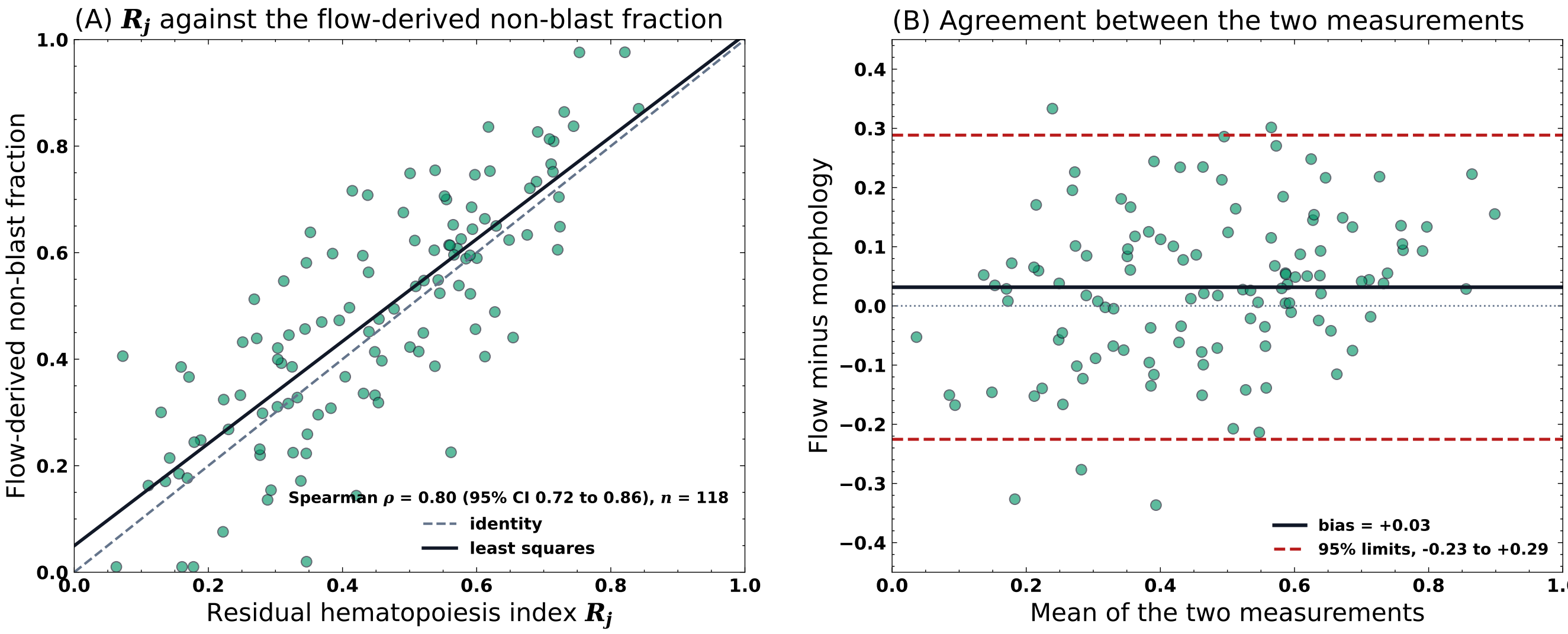

Figure S5. Agreement between the residual hematopoiesis index and the flow-derived non-blast fraction in the 118 patients with retrievable routine diagnostic flow cytometry. (A) Scatter with the identity line and a least-squares fit; Spearman correlation 0.80 (95% CI 0.72 to 0.86). (B) Bland-Altman plot; bias $+0.03$ with limits of agreement from -0.23 to $+0.29$. The limits are wide because a Wright-Giemsa differential and a flow gate count different denominators and haemodilution of the aspirate affects the two differently. The two measurements covary without being interchangeable.

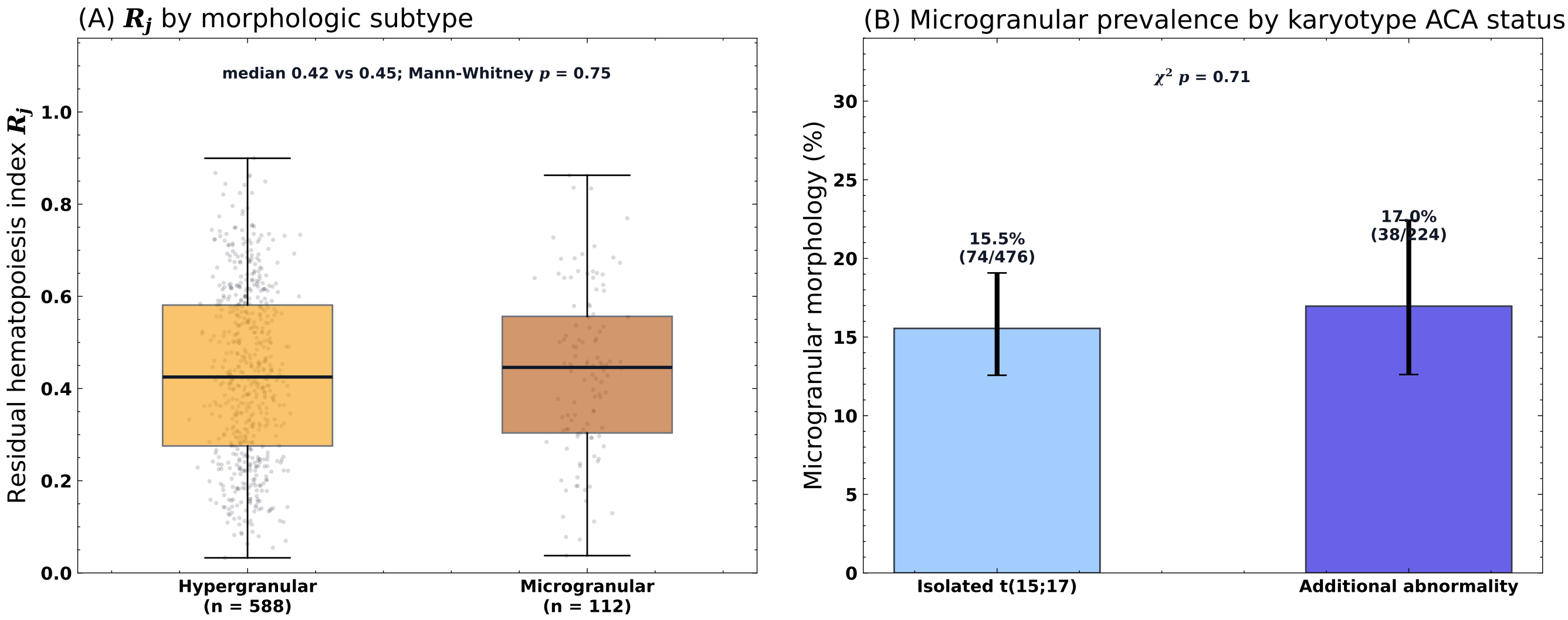

Figure S6. Tests of the classifier-error mechanism. (A) The residual hematopoiesis index by morphologic subtype: median 0.42 in hypergranular against 0.45 in micrgranular cases, Mann-Whitney $p = 0.75$. (B) Microgranular prevalence by karyotype ACA status, with Wilson intervals: 15.5% in patients with the isolated translocation against 17.0% in those with an additional abnormality, χ^2 $p = 0.71$. The mechanism requires microgranular cases to be over-represented among ACA-positive patients and the index to be inflated in them; neither condition holds in this cohort.

3 SUPPLEMENTARY TABLES

Tables S1 and S2 give the raw confusion-matrix counts from which every per-class metric in the main text derives. Tables S3 and S4 give the training configuration and the specimen acquisition detail needed to replicate the pipeline. Table S5 gives the three logistic models for differentiation syndrome, Table S6 the ascertainment and batch tests, Table S7 the literature search, and Table S8 the flow-cytometry subcohort.

Table S1. Raw un-normalized confusion matrix for the FISH fusion-pattern classifier, pooled out-of-fold over the five-repeat, five-fold cross-validation. Rows are true classes, columns predicted classes. Every precision, recall, and F1 in Table 4 of the main text is computed from these counts.

True \ Predicted	normal	typical	atypical	complex	pseudo-fusion	row n
normal	135	6	2	1	1	145
typical	8	607	5	5	2	627
atypical	8	3	31	1	0	43
complex	0	2	2	21	1	26
pseudo-fusion	0	1	1	0	7	9
column n	151	619	41	28	11	850

Table S2. Raw un-normalized confusion matrix for the seven-class marrow differential over the 7,500 hematopathologist-labelled cells, pooled out-of-fold. Rows are true classes, columns predicted classes, and the smear rows of Table 4 of the main text are computed from these counts. The exchange across the promyelocyte and maturing-myeloid boundary, which is the boundary that fixes the residual hematopoiesis index, is 78 true promyelocytes read as maturing myeloid (2.5% of 3,100) and 64 maturing myeloid cells read as promyelocytes (5.4% of 1,180).

True \ Predicted	pro	lym	mon	mat	ery	meg	pla	row n
promyelocyte / blast	2970	12	24	78	6	4	6	3100
lymphocyte	20	1364	24	2	10	0	30	1450
monocyte	22	20	435	32	2	2	7	520
maturing myeloid	64	10	34	1040	20	6	6	1180
erythroid precursor	30	26	2	18	806	0	8	890
megakaryocyte	10	2	3	8	0	143	14	180
plasma cell / other	12	26	6	4	8	1	123	180
column n	3128	1460	528	1182	852	156	194	7500

Table S3. Training configuration, hardware, and software. Values apply to every branch unless a branch is named.

Item	Value
Optimizer	AdamW, $\beta = (0.9, 0.999)$, weight decay 0.05
Learning rate	1×10^{-4} for fine-tuned encoders; 1×10^{-3} for classification heads
Schedule	cosine decay following a 5-epoch linear warmup
Batch size	32 images (FISH, karyotype); 256 cell crops (smear head)
Epochs	50 maximum
Early stopping	validation macro-F1, patience 10 epochs, best checkpoint restored
Gradient clipping	global norm 1.0
Precision	mixed precision (bfloat16 autocast)
Hardware	2 × NVIDIA RTX 4090, 24 GB each
Software	PyTorch 2.3.1, CUDA 12.1, Cellpose 3.0, Python 3.11
Training time	approximately 6 h per branch for the full five-fold, five-repeat schedule
Random seeds	five independent partitions, seeds fixed and recorded in the configuration files

Table S4. Specimen acquisition, instruments, and reagents. These fields support replication and the domain-shift discussion around the FISH-negative control images.

Item	Value
Smear stain	Wright-Giemsa, standardised laboratory protocol; two protocol epochs over the study period
Slide scanner	3DHISTECH Pannoramic 250 Flash III, Plan-Apochromat 40×/0.95 objective, 0.25 microns per pixel
Scanner camera	CIS VCC-FC60FR19CL colour line-scan camera, single focal plane
Smear quality control	fixed focus and staining criteria applied per field before inclusion
FISH probe	Vysis LSI PML/RARA dual-colour dual-fusion translocation probe (Abbott Molecular, 05J69-001), lots 1204871 to 1489233
FISH hybridisation	73 °C denaturation for 5 minutes, 37 °C hybridisation for 16 hours, DAPI II counterstain
Fluorescence microscope	Olympus BX53 epifluorescence microscope, UPlanFLN 60×/1.25 oil objective, DAPI, FITC (SpectrumGreen), and rhodamine (SpectrumOrange) single-band filter sets
Camera and exposure	Hamamatsu ORCA-Flash4.0 V3 monochrome sCMOS, 1×1 binning; 80 ms DAPI, 400 ms SpectrumGreen, 400 ms SpectrumOrange; fixed gain across the study period
FISH image capture	single representative field per patient, selected as described in the Methods of the main text
Karyotype banding	G-banding by trypsin-Giemsa
Band resolution	320 to 400 bands per haploid set
Karyotype imaging	Leica DM2000 LED brightfield microscope, HI PLAN 100×/1.25 oil objective, CytoVision capture station
Metaphase capture	raw, unarranged spread images, one per examined metaphase cell

Table S5. Logistic models for differentiation-syndrome occurrence. Model 1 is the primary adjusted model reported in the main text. Model 2 substitutes the continuous genetic-complexity score for the expert ACA flag. Model 3 is exploratory and is restricted to the 178 patients with FLT3-ITD testing.

Covariate	Odds ratio	95% CI	p
Model 1: expert ACA flag (n = 650, 189 events)			
Additional cytogenetic abnormality	1.07	0.74 to 1.55	0.72
$\log_{10}$ white-cell count	2.38	1.83 to 3.09	< 0.001
Platelet count, per 10×10^9/L	0.96	0.91 to 1.01	0.11
Age, per 10 years	1.09	0.98 to 1.21	0.11
Male sex	1.14	0.80 to 1.62	0.47
Corticosteroid prophylaxis	0.58	0.40 to 0.84	0.004
Model 2: continuous score (n = 650, other covariates as Model 1)			
G_j, per 0.1 unit	1.04	0.93 to 1.16	0.51
Model 3: exploratory, molecular subset (n = 178)			
Additional cytogenetic abnormality	1.11	0.61 to 2.02	0.73
PML::RARA bcr3 isoform	1.58	1.02 to 2.45	0.041
FLT3-ITD positive	1.74	1.05 to 2.89	0.032
$\log_{10}$ white-cell count	2.21	1.48 to 3.30	< 0.001

Table S6. Association between metaphase yield, scoreable nuclei per FISH image, accession year, and the two axes. These are the variables on the ascertainment and batch pathways.

Test	Spearman ρ	95% CI	p
Karyotype ACA score against metaphase yield L_j	0.11	0.04 to 0.18	0.004
Genetic-complexity score G_j against L_j	0.10	0.02 to 0.17	0.010
Residual hematopoiesis index R_j against L_j	0.14	0.07 to 0.21	< 0.001
FISH atypicality score F_j against nuclei per image	0.04	-0.03 to 0.12	0.24
R_j against nuclei per image	0.02	-0.05 to 0.10	0.53
R_j against accession year	-0.02	-0.09 to 0.05	0.60
R_j across accession years (Kruskal-Wallis)	—	—	0.57
R_j by staining protocol epoch (Mann-Whitney)	—	—	0.61

Table S7. Literature search supporting the statement that no published model reads two or more of cytomorphology, FISH, and karyotype images in the same patients for acute promyelocytic leukemia.

Item	Value
Databases	PubMed, Embase, Web of Science, IEEE Xplore
Date range	1 January 2015 to 31 December 2025
Last run	15 March 2026
Disease terms	"acute promyelocytic leukemia" OR APL OR "PML-RARA" OR "PML::RARA"
Modality terms	FISH OR karyotype OR cytogenetic OR morphology OR smear OR cytomorphology
Method terms	"deep learning" OR "machine learning" OR "artificial intelligence" OR CNN OR transformer
Records screened by title and abstract	1,284
Full-text records assessed	41
Eligible studies	0
Eligibility criterion	a model trained on two or more of the three image types in the same patients

Table S8. Diagnostic flow-cytometry subcohort. The non-blast fraction used as the external comparator for the residual hematopoiesis index is the proportion of nucleated events falling outside the abnormal promyelocyte and blast gate.

Item	Value
Patients with retrievable diagnostic flow cytometry	118 of 700
Specimen	the same diagnostic bone marrow aspirate
Gating	CD45 against side scatter, with the abnormal promyelocyte and blast population identified on CD45-dim, high side scatter
Antibody panel	CD3, CD19, CD34, CD117, CD13, CD33, myeloperoxidase
Derived quantity	fraction of nucleated events outside the abnormal promyelocyte and blast gate
Agreement with R_j	Spearman 0.80 (95% CI 0.72 to 0.86)
Bland-Altman bias	$+0.03$, limits of agreement -0.23 to $+0.29$
Primary association within this subcohort	Spearman 0.27 (95% CI 0.09 to 0.43)

REFERENCES

Schlichtkrull, M., Kipf, T. N., Bloem, P., van den Berg, R., Titov, I., and Welling, M. (2018). Modeling relational data with graph convolutional networks. In *The Semantic Web (ESWC 2018)*. 593–607. doi:10.1007/978-3-319-93417-4_38
